# Lipopolysaccharide-Induced Neuroinflammation as a Bridge to Understand Neurodegeneration

**DOI:** 10.3390/ijms20092293

**Published:** 2019-05-09

**Authors:** Carla Ribeiro Alvares Batista, Giovanni Freitas Gomes, Eduardo Candelario-Jalil, Bernd L. Fiebich, Antonio Carlos Pinheiro de Oliveira

**Affiliations:** 1 Department of Pharmacology, Universidade Federal de Minas Gerais, Av. Antonio Carlos 6627, Belo Horizonte 31270-901, Brazil; cacaribeiro@gmail.com (C.R.A.B.); gvnngomes@gmail.com (G.F.G.); 2 Department of Neuroscience, University of Florida, Gainesville, FL 32610, USA; ecandelario@ufl.edu; 3 Neuroimmunology and Neurochemistry Research Group, Department of Psychiatry and Psychotherapy, Medical Center–University of Freiburg, Faculty of Medicine, University of Freiburg, D-79104 Freiburg, Germany

**Keywords:** lipopolysaccharide, inflammation, neurodegeneration, Alzheimer’s disease, Parkinson’s disease, amyotrophic lateral sclerosis, Huntington’s disease

## Abstract

A large body of experimental evidence suggests that neuroinflammation is a key pathological event triggering and perpetuating the neurodegenerative process associated with many neurological diseases. Therefore, different stimuli, such as lipopolysaccharide (LPS), are used to model neuroinflammation associated with neurodegeneration. By acting at its receptors, LPS activates various intracellular molecules, which alter the expression of a plethora of inflammatory mediators. These factors, in turn, initiate or contribute to the development of neurodegenerative processes. Therefore, LPS is an important tool for the study of neuroinflammation associated with neurodegenerative diseases. However, the serotype, route of administration, and number of injections of this toxin induce varied pathological responses. Thus, here, we review the use of LPS in various models of neurodegeneration as well as discuss the neuroinflammatory mechanisms induced by this toxin that could underpin the pathological events linked to the neurodegenerative process.

## 1. Introduction

Neurodegenerative diseases are devastating conditions for which there is no cure so far. In general, the mechanisms involved in disease onset and development are still poorly understood. Therefore, increasing efforts are being made to better comprehend their pathogenesis. Among the different factors involved in these conditions, inflammation is considered a key contributor. Several lines of experimental evidence have demonstrated that neuronal cell death may induce an inflammatory process, and inflammation by itself may lead to cell death [1]. Thus, it is necessary to induce inflammation in models of neurodegeneration in order to evaluate its intricate consequences. 

Induction of inflammation may be achieved in different manners, and lipopolysaccharide (LPS) is an important tool for this purpose. LPS is a molecule present in the outer membrane of Gram-negative bacteria. Its main target is the toll-like receptor (TLR) 4, although it is known to act on other receptors [2,3,4]. The activation of TLR4 by LPS recruits a series of downstream adaptors, such as myeloid differentiation primary response protein 88 (MyD88), TIR-domain-containing adaptor-inducing interferon-β (TRIF) and TRIF-related adaptor molecule (TRAM), which are crucial for the signaling of the receptor [5,6]. The recruitment of these adaptors can further activate downstream pathways which culminate in the activation of transcription factors, which, in turn, induce a plethora of pro-inflammatory genes [6,7,8]. The TLR4 signaling pathway has been fully reviewed elsewhere [9].

Although most of the work in this field uses LPS in order to stimulate glial cells, mainly microglia, it is known that neurons also express TLR4. Indeed, activation of this receptor leads to the neuronal production of different inflammatory mediators [10,11,12,13].

LPS is used in a variety of in vivo and in vitro protocols. This compound not only is used to stimulate cell cultures, but also is injected either in the central nervous system (CNS) or in the periphery by single or multiple injections. Thus, its effects may vary according to the experimental protocol. Therefore, here, we review the various protocols that use LPS in order to provide an overview of the current state of the art. We also discuss the advantages and limitations of the LPS models used to understand the complex molecular and cellular mechanisms underlying the neuroinflammatory process associated with neurodegeneration. 

## 2. LPS-Induced Inflammation in Models of Alzheimer’s Disease

Alzheimer’s disease (AD) is the most common neurodegenerative disorder worldwide, and its main clinical manifestation is progressive dementia [14]. It is characterized by the inability to form new memories, reflecting the dysfunction of the episodic memory system [15,16]. AD is associated with neuropathological changes such as the formation of tau aggregates seen as intraneuronal neurofibrillary tangles and the presence of extracellular amyloid-beta (Aβ) plaques [17,18]. It was demonstrated that activated microglia are present in regions of the brain where there are Aβ deposition and neuronal loss, which culminates in memory impairment. Published data showed that chronic LPS administration produced impaired spatial memory in Sprague Dawley [19] and Fisher rats [20]. 

Neuroinflammation frequently precedes the development of neurodegenerative pathologies such as AD [21] and is one of the pathogenic factors for neurodegeneration [22]. Significant studies from basic cellular neuroscience and human genetics support the important role of inflammation in the pathogenesis of AD [23,24,25]. The myeloid cells of the CNS, microglia, can be beneficial and detrimental to AD pathogenesis, since they can degrade amyloid plaques and promote neurotoxicity due to excessive inflammatory cytokine release [23,26]. LPS-induced inflammation is used in experimental in vitro and in vivo models of neuroinflammation and has been shown to also promote amyloid deposition in vivo [27,28].

Some studies have associated AD neuropathology with LPS levels in the brain. The presence of LPS and Aβ1–40/42 in amyloid plaques in gray and white matter of AD brains has been demonstrated [29]. Another study showed that LPS is abundant in the neocortex and hippocampus of AD-affected brains and that there is a strong adherence of LPS to the nuclear periphery in AD brain cell nuclei [30]. Finally, LPS was also found in lysates from the hippocampus and superior temporal lobe neocortex of AD brains [31]. The role of LPS in the development of AD is reviewed by [32,33]. 

In this context, experimental models using LPS could serve as a link between neuroinflammation and AD and are useful to understand the disease process and some events that occur in human AD.

### 2.1. Contribution of Central LPS Injection Models to Our Understanding AD Pathology

Animals can respond to LPS stimuli differently depending on age and species. In addition, the source of the stimulus, the dose, the route, and the duration of the administration used in each study may also influence the outcome [34]. LPS injection in different regions of the CNS leads to a variety of responses in animals. In this section, we will discuss the data obtained from LPS-induced inflammation in the CNS associated with AD.

Single intracerebroventricular (i.c.v.) injections of LPS resulted in increased levels of interleukin-1β (IL-1β) in the brainstem and diencephalon of rats 2 h after injection, and in all the brain regions, except cerebellum, 6 h after injection [35]. Besides, the induction of IL-1β mRNA in the nucleus basalis magno-cellularis and hippocampus was observed, as well as the presence of mRNA for tumor necrosis factor-α (TNF-α) in the nucleus basalis magno-cellularis [19]. 

Microglia play an important role in immune defense and inflammatory responses in the CNS [36]. When microglia are exposed to stimulatory molecules such as LPS, their receptors such as TLRs recognize LPS, inducing a series of intracellular signaling pathways [37,38]. Activation of microglia and astrocytes was observed after both single i.c.v. LPS injection and chronic LPS injection in the 4th ventricle with osmotic pumps [19,39]. In addition, single intrahippocampal LPS injections produced elevations of glial fibrillary acidic protein (GFAP) after 24 h [40]. On the other hand, 28 days after a single intrahippocampal LPS injection, chronic microglial activation was observed, marked by the increase of CR3 and CD45 in the mouse hippocampus [40]. These are important findings, since glial activation after intrahippocampal LPS injection has been related to AD-like amyloidogenic axonal pathology and dendritic degeneration [41]. Chronic i.c.v. administration of LPS induced β-amyloid precursor protein (β-APP) mRNA in the nucleus basalis magno-cellularis of rats [19]. In a marmoset monkey model, LPS co-injected with Aβ fibrils in frontal, sensorimotor, and parietal cortices accelerated the amyloidosis process, with all monkeys showing an early AD immune blood cell expression profile of the apoptosis receptor CD95 [42], suggesting a potential synergic action.

In many neuroinflammatory conditions, including in mouse models of AD, microglia activation and infiltration of peripheral immune cells are found in the brain parenchyma [43]. Microglia activation is also associated with hyperphosphorylation and aggregation of the protein tau, another important AD marker. Single intrahippocampal injection of LPS enhanced tau phosphorylation by about 2.5-fold via microglial activation in rTg4510 mice, which carry a mutant tau [44]. 

Microglia response after stimulation with LPS may differ between transgenic and non-transgenic mice. Although microglia in 12-month-old non-transgenic mice showed a stronger response to LPS than in 2-month-old mice of the same strain, microglia in transgenic APP/PS1 mice exhibited diminished immune response to LPS during aging. Microglial TLR4 signaling was altered in transgenic mice, suggesting that changes in TLR4 signaling may have impaired the Aβ clearance capacity of microglia [45]. In Tg2576 mice, which express a mutant form of APP, a single LPS intrahippocampal injection reduced hippocampal Aβ levels in a time- and glial activation-dependent manner [46,47]. Another study showed that intrahippocampal LPS injection increased by about sixfold the bone marrow cells recruitment from the periphery and reduced Aβ clearance in bone marrow-transplanted AD transgenic mice [48].

### 2.2. Systemic LPS Challenge Models Utilized to Understand AD Pathology

Systemic inflammation may affect the brain. Cytokines, such as IL-1β, IL-6, and TNF-α, produced by a systemic inflammatory response, can reach the CNS through the blood circulation [49]. The intraperitoneal (i.p.) injection of LPS, for example, leads to the detection of IL-1 in the plasma and brain regions [35]. The levels of TNF-α, IL-1α, IL-1β, and IL-6 mRNAs were increased in the hippocampus and cerebral cortex of mutated presenilin (PS) 1 transgenic mice compared to wild-type mice after i.p. injection of LPS [50]. The increase in mRNAs levels of IL-1β and IL-6 due to a single LPS i.p. injection was associated with changes in APP expression in the cerebellum of Staggerer mutant mice, which show a severe Purkinje cell deficiency in the cerebellum, whereas the cerebral cortex is not affected [51]. Similarly, a single LPS injection increased IL-1β and TNF-α by about twofold in cortices and hippocampi of aged Tg2576 mice 1, 2, 4, and 6 h after stimulus [52] and increased the blood and brain levels of IL-1β, IL-6, and TNF-α in Sprague Dawley rats [53]. In addition, in a model of LPS-induced cognitive impairment in rats, TNF-α levels were increased by about 1.6-fold in the hippocampus and frontal cortex after 7 days of a single LPS injection. Interestingly, TNF-α and IL-18 were increased in the same areas after 10 months of a single LPS injection [54]. TNF-α plays an important role in the induction of inflammatory processes, being recruited after the LPS stimulus and inducing the production of pro-inflammatory cytokines, which are involved in the pathophysiology of neurodegeneration. IL-18 might act later, when the disease is already established, participating in the progression of neurodegeneration and cognitive dysfunction. 

Systemic administration of LPS also induces microglial activation. A single LPS injection increased microglial density in Sprague Dawley rats [53]. The brain metabolic response to LPS-inducing microglial activation was studied using magnetic resonance spectroscopy. Intraperitoneal injection of LPS also increased the number of Iba-1(+) microglia and induced Aβ(1–16)(+) neurons in the hippocampus in C57/CJ mice [55]. 

LPS has been used in different studies to stimulate the production of β-APP. Peripheral stimulation with LPS induced an increase in IL-1β and IL-6 mRNAs, followed by changes in the expression of APP isoforms in the cerebellum [51]. LPS administration for 7 days increased Aβ 1–42 cerebral expression and triggered AD-like neuronal degeneration [56]. On the other hand, chronic LPS administration increased by about twofold the number of Aβ and APP immunoreactive neurons in the neocortex of APPswe mice [28]. A similar increase in Aβ was seen in the hippocampus of EFAD mice (a model that expresses human APOE3 or APOE4 and overproduces human Aβ42) [57] and in the hippocampus, cortex, and amygdala of APPswe mice receiving chronic LPS administration [28]. In all these transgenic models, increased Aβ neuronal immunoreactivity was associated with an elevated number of F4/80-immunoreactive microglia [28] and an increase in the 6E10-immunoreactive protein, which contains Aβ fragments [58]. Repeated LPS systemic injections (three or seven times) promoted Aβ 1–42 accumulation in the hippocampus and cerebral cortex of ICR albino mice, as a result of an increase in beta- and gamma-secretase activities as well as in the activation of astrocytes in parallel to cognitive impairment [59]. A reduction of Aβ accumulation in hippocampus, cortex, and amygdala was demonstrated by chronic LPS injection in 3xTgAD mice, which exhibit both Aβ and tau pathologies, in combination with an inhibitor of soluble TNF-α signaling [58]. In addition, young and old transgenic mice showed an increase in Aβ 1–40 in the cortices between 4 and 6 h after LPS administration, which returned to baseline 18 h after a single injection [52]. However, LPS once a week for 13 weeks ameliorated amyloid pathology in the neocortex of APP_SWE_/PS11_∆E9_ mice [60], which was associated with increased aggregation of activated microglia around the Aβ deposits and by CNS myeloid cells inducing Aβ clearance pathways and elevated levels of the lysosomal protease cathepsin Z as well as clusterin [60]. Contradictory data suggest that there are differences in the amyloid production and that the accumulation depends on the degree of severity of inflammatory stimuli and the animal model used to evaluate the consequences of LPS injection. Indeed, it has been demonstrated that LPS-induced inflammation can contribute to the progression of a series of neurodegenerative processes [61,62]. On the other hand, immune system stimulation with low doses of LPS can induce the activation of cells that act on the resolution of the pathology in neurodegeneration [63,64,65].

A deficiency in Aβ clearance due to an impairment of the blood–brain barrier (BBB) has been associated with AD development [66]. In this way, the integrity of the BBB is important, since Aβ clearance ameliorates AD neuropathology [67]. Besides, an association between AD and lipoprotein receptor-related protein-1 (LRP-1)—a member of the low-density lipoprotein receptor family—has been demonstrated to participate in Aβ metabolism [68]. In this sense, some studies demonstrated that LPS induced an Aβ transport dysfunction at the BBB dependent on LRP-1 [67,69]. Repeated i.p. injection of LPS altered the BBB transport of Aβ by increasing the brain influx and decreasing the efflux of the peptide. In addition, LPS also increased the expression of neuronal LRP-1, which can be responsible for the increased production and accumulation of Aβ in the brain [69]. Similarly, another study showed a decrease in Aβ efflux by LPS-induced dysfunction of LRP-1 at the BBB [70]. A disruption of the BBB by LPS was observed in aging 5XFAD mice, which overexpress both mutant human APP and presenilin 1. On the other hand, inflammation induced by LPS may also be an interesting tool for the crossing of drugs through the BBB. Indeed, Barton et al. (2018) demonstrated that LPS may disrupt the BBB in 5XFAD mice, which improved the delivery of small molecules, such as thioflavin S, to the brain [71]. Therefore, the neuroinflammatory process could also play an important role in the pathophysiology of AD by disrupting the BBB and impairing the removal of Aβ from the brain, as well as in facilitating a pharmacological treatment.

Increased levels of Aβ induced by LPS can promote tangle formation [53]. In fact, single LPS injection increased the levels of soluble Aβ and phosphorylated tau in the brain of rats [53] and mice [72]. Acute systemic LPS administration enhanced tau phosphorylation in wild-type and corticotropin-releasing-factor-receptors (CRFR)-deficient mice, which was associated with the activation of glycogen synthase kinase-3 (GSK-3) and cyclin-dependent kinase-5 (CDK5) [73]. Similarly, tau hyperphosphorylation in 3xTgAD mice was also mediated by the activation of CDK5 after chronic LPS administration [74]. 

Cognitive deficits were shown by studies using single LPS i.p. administration in rats [56] and by repeated LPS injection in EFAD mice [57]. Besides the cognitive impairment and the increase by more than tenfold in the levels of Aβ with a single i.p. administration of LPS, the elevation of nitric oxide (NO) concentrations and the overexpression of *N*-methyl-d-aspartate receptor subunit 2B (NMDAR2B) in the brain were described [75]. 

Finally, neuroinflammation is regulated through the cholinergic anti-inflammatory pathway by the α7 nicotinic acetylcholine receptor (α7 nAChR), involved in regulating cognitive functions and inflammatory reactions. It was demonstrated that systemic LPS injection in mice decreased α7 nAChR in the brain [76,77]. Thus, this may be another mechanism by which LPS induces neuroinflammation and cognitive impairment in models of AD.

The data presented in Section 2 demonstrate the large number of studies using LPS to induce neuroinflammation in models associated with AD. There is enough evidence to support the singular role of neuroinflammation in neurodegeneration in addition to the importance of animal models to study Aβ accumulation and tau hyperphosphorylation. In summary, it can be assumed that LPS injection models mimic memory loss and the neuropathology observed in AD. All these studies help understand the role of neuroinflammation in the progression of AD.

## 3. LPS-Induced Models of Parkinson’s Disease

Parkinson’s disease (PD) is the second most prevalent neurodegenerative disorder [78], and its neuropathology is characterized by the degeneration of dopaminergic neurons in the substantia nigra (SN), followed by the loss of axonal projections to the striatum, resulting in malfunction of the dopaminergic system [79,80]. Dopaminergic dysfunction manifests in the characteristic motor disabilities found in the disease, such as tremor, rigidity, bradykinesia, postural, and gait abnormalities [81,82]. Cytoplasmic inclusions, known as Lewy bodies, which are essentially constituted by protein deposits of α-synuclein [83], are the main hallmark feature of PD. Although the etiology of PD is not well known, it has been described that inflammation contributes to PD progression and is an important factor related to neuronal loss [84,85,86].

To focus on the potential role of inflammation in PD, several LPS-induced Parkinson models have been validated and used. Different routes of injection, doses, species models, and sources of endotoxin are described. In the following two sections, we will present the main contributions of LPS-induced models to providing more insights into the pathophysiology of PD.

### 3.1. Contribution of Central LPS Injection Models to the Elucidation of PD Pathology

Part of the knowledge about the involvement of neuroinflammation in PD was obtained from models of central injection of LPS into the SN or striatum (ST). Both models of injection can induce the dopaminergic neurodegeneration and motor symptoms characteristic of the disease. A first intranigral LPS injection was established in 1998, inducing microglial activation after 2 days, followed by a reduction in dopamine levels in the SN and ST and a decrease in tyrosine hydroxylase (TH) activity up to at least 21 days [87]. Later studies tested the impact of LPS injection on dopaminergic neurodegeneration and microglial activation. A permanent dopaminergic neuron loss after a single LPS injection into the SN was observed up to one year after the injection. Neuronal loss was associated with a strong macrophage/microglial reaction in the SN [88,89,90,91]. The inflammatory involvement was supported by the use of drugs that reduce the effects mediated by microglia. Intranigral or systemic administration of naloxone, an opioid receptor antagonist, prevented neuronal loss induced by local LPS injection in the SN [92]. Eight or 15 days of systemic dexamethasone administration prevented the reduction of TH activity and TH immunostaining induced by intranigral LPS injection, suggesting a reduction of dopamine dysfunction in addition to the reduction of microglia activation [93]. This first set of investigations supported the idea that microglia-mediated neuroinflammation plays an important role in the neurodegenerative process of PD.

LPS injection into the CNS clearly increased the expression of inflammatory mediators in the brain. Elevated levels of TNF-α and IL-6 in the SN and elevated IL-6 in the ST 90 minutes after intranigral LPS injection were found in C57BL/6 mice. Interestingly, the authors observed a 29-fold and 36-fold increase in peripheral circulating levels of TNF-α and IL-6, respectively. The peripheral levels of IL-2 and IFN-γ were also increased at day 7 post-injection, whereas no changes in these inflammatory mediators were detected in the SN. These effects were accompanied by increased CD11b immunostaining in the SN [94], which suggests an ongoing microglial activation and neuroinflammation. A comparable cytokine profile was observed after intranigral LPS injection [95,96]. In a chronic LPS injection approach that mimics the early stages of many chronic neurodegenerative diseases, the injection of LPS into the 4th ventricle for 21 or 56 days induced different responses that depended on the animal’s age and the stimulus duration [97]. Gene expression and protein levels of both pro- and anti-inflammatory parameters were upregulated in the brainstem, with IL-1β, TNF-α, TGF-β, and CX3CR1 being the most important ones. Importantly, these changes in cytokine expression and loss of TH-positive neurons were more pronounced in middle-aged and aged rats compared to young rats.

A recent study evaluated the time-dependent expression of pro- and anti-inflammatory cytokines after intranigral LPS injection in adult Wistar rats. The levels of TNF-α and IL-1β mRNA were significantly increased at early time points, with a maximum after 5 h (~threefold and ~fourfold increase, respectively), while IL-6 mRNA levels were maximal after 8 h (about fivefold increase). Interestingly, IL-1β mRNA levels remained significantly increased up to 168 h after LPS injection [98]. On the other hand, anti-inflammatory mRNA expression was altered only at late time points (after 24 h and 168 h for IL-10 and IL-4, respectively). These effects were followed by microglial and astrocytic activation and dopaminergic neurodegeneration in the SN [98]. Moreover, the changes in the inflammatory mediator profile were in line with the increased expression of nuclear factor kappa B (NF-κB) after intracerebral LPS injection, which can lead to a significant increase in the transcription of pro-inflammatory cytokines (e.g., TNF-α and IL-1β) [99,100,101,102]. Furthermore, LPS intrastriatal injection caused an oxidative stress response and apoptosis, which are strongly associated with the activation of TLR/NF-κB signaling and the inhibition of the anti-oxidant Nrf/HO-1 pathway [103]. These data suggest that the LPS injection models induce an acute initial pro-inflammatory profile and that the neuronal degeneration process in the SN and ST are mediated by these inflammatory mediators, which are therefore crucial for the progression of the pathology.

Mitochondrial dysfunction is also associated with neuronal cell death in the pathogenesis of PD [104,105]. In this way, LPS injection models can contribute to evaluate possible impairments in mitochondrial activity to elucidate their impact in the pathophysiology of PD. Intrastriatal LPS injection induced changes in the mitochondrial respiratory chain, evidenced by increased levels of oxidative stress markers including protein carbonyls, 4-hydroxynonenal (4-HNE), and 3-nitrotyrosine (3-NT), and caused structural modifications in the mitochondrial cristae, leading to energy dysfunction and neuronal loss in the striatum [106]. Mitochondrial dysfunction was also supported by increased PPAR-γ, UCP2, and mitoNEET expression—three proteins involved in energy metabolism—in the SN [107]. Moreover, intrastriatal injection of LPS induced extensive S-nitrosylation/nitration of the mitochondrial complex prior to dopaminergic neuronal loss [108]. Related to this previous finding, inhibition of inducible nitric oxide synthase (iNOS) by l-N6-(l-iminoethyl)-lysine reduced mitochondrial injury and dopaminergic degeneration induced by LPS injection into the SN, indicating that iNOS-derived NO is associated with mitochondrial dysfunction. iNOS activation is mediated by p38 MAP kinase, and cell death was reduced by the inhibition of p38 [109].

Intranigral LPS injection upregulated iNOS expression (~twofold) and elevated total reactive oxygen species (ROS) production (~twofold) and NADPH oxidase activity (~fivefold) [99]. Supranigral administration of LPS induced an intense expression of NADPH-diaphorase and iNOS-immunoreactivity in macrophage-like cells, followed by an important decrease of tyrosine hydroxylase-positive neurons [110]. Pre-treatment of animals with the iNOS inhibitors *S*-methylisothiourea or L-NIL prevented dopaminergic neuronal loss, suggesting that NO mediates the neurodegeneration observed in the LPS-induced PD model [110,111]. Moreover, a single intrastriatal LPS injection was found to be associated with increased striatal cyclooxygenase-2 (COX-2) and iNOS expression three days post-injection and, in the SN, dopaminergic neuronal loss and an increase in microglia activation were observed seven days post-injection [102,112]. Furthermore, a two-week intracerebral infusion of LPS (5 ng/h, delivered using osmotic minipumps) induced a rapid activation of microglia that reached a plateau at the end of the treatment, followed by a delayed and gradual loss of nigral dopaminergic neurons starting between four and six weeks after treatment [90], suggesting that the initial activation of the immune response preceded neuronal loss. 

In line with the studies described above, LPS injection was shown to alter iron and ferritin levels in glial cells of the SN of rats, which was associated with 1.5-fold and 2.5-fold decreases in TH expression in the globus pallidus [113] and in the striatum [107], respectively. It was also demonstrated that iron chelation with desferrioxamine attenuated behavior deficits, neuronal loss of dopaminergic neurons, and striatal dopamine (DA) reduction induced by intrastriatal LPS injection in C57BL/6 mice [114]. The data from studies involving mitochondrial activity and the NO cascade suggest that oxidative stress and mitochondrial dysfunction are important in PD progression, including dopaminergic dysfunction and α-syn accumulation, which can promote neurodegeneration in SN and deficits in locomotor activity.

Familial PD cases account for 10% of total cases of the disease [115,116], but the molecular mechanisms involved in the onset of familial forms still need to be elucidated. Neuroinflammation can also contribute to the progression of the genetic forms of PD. Mutations in the gene encoding for leucine-rich repeat kinase 2 (LRRK2) are associated with familial PD [117], with an increased lifetime risk for developing sporadic PD [118]. In an intranigral LPS-injection model of neuroinflammation, a robust induction of LRRK2 in microglial cells was observed [119]. In addition, injection of LPS into the SNpc of LRRK2 KO rats resulted in less pronounced TH-positive neuron loss, microglial activation, and elevated level of iNOS compared to wild-type rats [120]. A morphological evaluation revealed that the fractal dimension—a quantitative computer-based analysis for cell complexity evaluation—of Lrrk2^−/−^ microglia was significantly lower than that of Lrrk2^+/+^ cells in the striatum injected with LPS [121]. The expression of the protein deglycase DJ-1 (PARK7)—whose gene is related to autosomal recessive forms of PD [122,123]—can be also impacted by inflammatory challenges. It is known that mutations in the *PARK7* gene are associated with loss of dopaminergic neurons due to the upregulation of inflammatory mediators within the SN, which was demonstrated by LPS intranigral injection in *PARK7* DJ-1^−/−^ KO mice [124]. These data suggest that inflammatory events that occur throughout life can contribute to the progression of diseases related to autosomal dominant or autosomal recessive mutations, as shown by results from several experimental investigations. 

Experimental data obtained from local injections of LPS into the CNS have contributed to the elucidation of the pathophysiology of PD, including the familial form of the disease. In the next section, data from models that used systemic LPS challenges will be presented. Inflammatory processes in the periphery can induce both acute and adaptive responses and contribute to deleterious effects on the CNS because of the action of inflammatory mediators from the periphery that are released into the brain [35,125,126]. Thus, peripheral inflammatory challenges can contribute to a better understanding of the crosstalk between inflammation, neuroinflammation, and basic aspects involved in neurodegenerative conditions.

### 3.2. Contribution of Systemic LPS Challenge Models to the Elucidation of PD Pathology

Systemic LPS challenge is another model to elucidate neuroinflammation in PD. Single or multiple LPS injections were used to provide valuable insights into the potential pathogenesis of PD. Molecular and cellular alterations were found after LPS i.p. injection in C57BL/6 mice. Brain TNF-α was elevated for up to 10 months after LPS injection, suggesting a sustained brain TNF-α overproduction that was parallel to microglial activation and delayed and progressive loss of nigral TH-positive neurons [127]. Extensive neuronal loss, decline in dopamine levels, glial activation, altered cytokine profile on SN, and deficits in locomotor behavior were also observed after four consecutive days of peripheral LPS injections [128]. Additionally, authors described a time-course shift of cytokine profiles from pro- to anti-inflammatory. Five to 19 days after exposure, pro-inflammatory mediators were predominant, in parallel with neuronal loss, while anti-inflammatory molecules were predominant between days 19 and 38 post-injection. Interestingly, a single dose of LPS failed to elicit neuroinflammatory responses in female mice [129]. On the other hand, i.p. injections of LPS for five weeks (one injection per week) or for five months (one injection per month) could cause loss of TH-positive neurons in the SN 9 and 20 months after injection, respectively. In addition, motor impairment as well as a more intense immuno-staining for α-syn and inflammatory markers were observed [129]. The augmentation of protein aggregation and nigral inflammatory process was also observed in a study that compared the effect of LPS i.p. injections in wild-type mice and in transgenic mice that overexpressed α-syn. It was demonstrated that transgenic mice, but not wild-type mice, developed a delayed chronic and progressive degeneration of nigral TH-positive DA neurons, with a more prominent effect five months after LPS injection. In addition, transgenic mice treated with LPS accumulated ~1.3-fold more α-syn aggregation than non-treated or wild-type mice [61]. The synergic impact of α-syn and inflammation on the BBB was also evaluated. Knockout mice for α-syn (Snca^−/−^) were subjected to LPS exposure, and it was noticed that α-syn did not alter BBB permeability in the absence of an LPS challenge. However, LPS injection induced significant augmentation in BBB permeability in normal wild-type, but not in knockout, mice [130].

α-Syn overproduction and its accumulation appear to be associated with an impaired autophagy process. Alterations in autophagic protein levels were noticed after LPS injection. Early-period evaluations (starting at day 1) revealed increased levels of microtubule-associated protein 1 light chain 3-II (LC3-II) and histone deacetylase (HDAC) 6. On the other hand, p62 level remained increased until late stages (from one day to seven months after LPS injection). A significant increase in α-syn protein in the midbrain was also found in this study, suggesting that LPS might cause an impairment of α-syn clearance [131]. Therefore, peripheral inflammatory stimuli may be an important synergic factor for α-syn-induced pathology in PD, and autophagy activity failure might be involved in the increased protein aggregation induced by the LPS challenge.

The participation of NO, oxidative stress, and mitochondrial impairment was also investigated after peripheral LPS injection. Wide ultrastructural changes were observed in SN neuronal cells, including axons alterations, the swelling of mitochondria and the Golgi complex, and the presence of autophagolysosomes, lysosomes, and dense bodies in the cytoplasm. In addition, the presence of apoptotic cells and glial activation was also observed [132]. iNOS induction was observed at the initial phase of response to the peripheral LPS injection [128]. NOS activity in the midbrain and in SN was increased 6 h after LPS treatment [132]. Furthermore, exposure of C57BL/6 mice to LPS resulted in a large increase in NOX2 mRNA expression in the midbrain 24 h after exposure, associated with a rapidly increased ROS production at 1 and 24 h [133]. Treatment of NOX2^−/−^ mice with LPS demonstrated the contribution of this mediator to the pathology-associated neuroinflammation, since knockout mice presented less dopaminergic neuronal loss and reduction of microglial activation in the midbrain after LPS i.p. injection [133]. It was also observed that, despite a lack of changes in caspase-3 activity, LPS injection induced apoptosis-inducing factor (AIF) translocation from the mitochondria to the nucleus. Moreover, iNOS and nNOS (the neuronal constitutive form of NOS) inhibition prevented LPS-evoked release of AIF from the mitochondria, indicating that the increased synthesis of NO occurring in the brain during systemic inflammation might be responsible for the activation of apoptotic pathways [132]. Lastly, iNOS and NADPH oxidase inhibition was also associated with the reduction of chronic neuroinflammation and prevented α-syn pathology and dopaminergic neuronal loss in transgenic mice that overexpressed human A53T mutant α-syn submitted to LPS i.p. injection [61].

The role of oxidative stress in PD seems to be age-dependent. The upregulation of pro-oxidant and inflammatory factors was shown in the midbrain of aged C57BL/6 mice submitted to acute i.p. injection of LPS, compared with young mice injected with LPS [134]. In addition, LPS induced a more severe loss of DA neurons in aged female C57BL/6 mice. The upregulation of TLR2, p-NF-κB-p65, IL-1β, TNF-α, iNOS, and gp91phox was also associated with aging [135]. These data indicate an important aspect of aging in the neuroinflammatory process found in PD and evidence the overexpression and overproduction of factors associated with oxidative stress in aged rodents injected with LPS.

In summary, SN and ST are highly sensitive and strongly affected by systemic LPS administration. Findings from studies using peripheral LPS injection can contribute to the understanding of the progression of PD, in particular, to the comprehension of its neuroinflammatory aspect. 

## 4. LPS Models to Understanding Inflammatory and Neuroinflammatory Aspects in Amyotrophic Lateral Sclerosis and Huntington’s Disease

### 4.1. Amyotrophic Lateral Sclerosis

Amyotrophic lateral sclerosis (ALS) is a neuromuscular disorder associated with the voluntary motor system, characterized by the progressive degeneration of anterior-lateral horn spinal cord motor neurons leading to weakness and eventual death of the affected individuals by paralysis in a few years [136,137]. The degenerating neurons present an abnormal accumulation of cytoplasmic inclusions containing ubiquitinated proteins [138]. A role of inflammation in the pathogenesis of ALS has been suggested [139,140]. In this sense, LPS-induced inflammation may contribute to the knowledge of the involvement of neuroinflammation in the pathophysiology of ALS. 

The overexpression of mutant copper, zinc superoxide dismutase (SOD) in mice is utilized as a model of ALS, inducing severe hind limb motor deficits in animals [141]. These G93A-SOD1 mice were challenged with LPS to evaluate the possible impact of systemic inflammation in this model. LPS injection increased the nuclear expression of the transcription factor CCAAT/enhancer binding protein δ (C/EBPδ), whose gene is associated with familial ALS, in the spinal cord of G93A-SOD1 mice [142]. Moreover, astroglial and microglial activation were also associated with LPS-induced inflammation in an ALS experimental model [142,143].

About 5% of ALS cases are familial forms of the disease [144]. TAR DNA-binding protein (TDP-43), a major component of cytoplasmic inclusions in sporadic and most familial ALS cases, appeared accumulated and aggregated in the cytoplasm of spinal motor neurons of TDP-43^A315T^ transgenic mice after chronic LPS administration [145]. 

However, there are only a few studies using LPS to induce inflammation in animal models of ALS, despite the knowledge about the role of immune and inflammatory components in this neurodegenerative disease [146,147]. More studies are necessary to clarify the gaps associated with this disorder.

### 4.2. Huntington’s Disease

Huntington’s disease (HD) is a neurodegenerative disease characterized by motor, cognitive, and behavioral dysfunctions [148,149]. HD is originated by an autosomal mutation that is characterized by an increase in the number of CAG repeats in the huntingtin (*HTT*) gene [150], resulting in the expansion of a polyglutamine tract in the resulting mutated HTT (mHTT) protein that is neurotoxic. mHTT aggregates are abundant in the nuclei and processes of neuronal cells and lead to several damages, including protein malformation, transcriptional dysfunction, irregular protein and vesicle transport, altered secretion of neurotrophic factors, and others [151,152,153,154]. The immune and inflammatory component has also been linked to the progression of HD. Changes in the cytokine profile were reported in the post-mortem brain [155] and in the plasma and serum of patients [156], and several lines of evidence of inflammation involvement have been provided by animal models [62,157,158,159].

Studies on the impact of inflammatory challenges in this neurodegenerative disease are rare. Peripheral injection of LPS enhanced some aspects of HD, such as microglial alterations and vascular dysfunction, as shown in 12-month-old YAC128 transgenic mice—a model that expresses human mutant huntingtin protein—challenged chronically (four months) with LPS. Changes were characterized by an increased number and morphological changes of microglia in the ST. Furthermore, an increased vessel diameter and wall thickness in the same region and disruption of the BBB permeability were observed [159]. These data indicate that LPS enhances the inflammatory response in this model of HD. Levels of proinflammatory cytokines after a single LPS i.p. injection were higher in the cortex and ST of brains obtained from Hdh^150Q^ mice (which carry 150 CAG repeats in the first exon of the endogenous gene) and R6/2 mice (which express exon 1 of the human *HD* gene with 150 CAG repeats) compared with wild-type animals [62]. The authors observed that LPS exposure caused an increased nuclear localization of p65—a NF-κB subunit—in both astrocytes and microglia in the cortex of R6/2 mice compared with wild-type mice, contributing to neuroinflammation. In addition, the levels of TNF-α remained elevated in brain, serum, and liver of the two HD mouse models after systemic LPS injection [62]. Thus, a peripheral inflammatory process contributed to the progression of HD and to a more prolonged neuroinflammation mediated by glial cells.

Interestingly, a sex-dependent response of HD R6/1 mice to an LPS single injection was demonstrated. Authors noticed that LPS-induced TNF-α expression was ~1.5-fold higher in the hypothalamus of female HD mice as compared with female wild-type mice. In contrast, LPS treatment induced an opposite effect in male HD subjects, with largely diminished TNF-α gene expression, compared with wild-type mice [160]. More lines of evidence are necessary for a better exploration of these sex-dependent aspects, but these observations might suggest differences in HD patients, depending on their gender. 

Nevertheless, chronically low-dose LPS injections activating the immune system showed a significantly prolonged survival of HD R6/2 animals, less pronounced body weight loss, and an attenuated clinical score of the clasping phenotype compared with wild-type animals treated with the endotoxin [65]. Therefore, the role of inflammatory processes in HD needs to be further elucidated, and the link between neuroinflammation and HD progression may be dependent on age, gender, and severity of the inflammatory challenge. 

As it can be observed by reading the reports mentioned above, different factors may be important for the outcome of the studies, which include the source of LPS, dose, route and scheme of administration. Therefore, we built tables (Table 1, Table 2, Table 3, Table 4, Table 5 and Table 6) that further detail all these differences that must be considered for the planning of an experimental protocol design. In the tables, only papers that provide full information about the type of LPS used were included.

## 5. LPS in Cell Culture Models 

The basic aspects of the neurodegenerative process were elucidated by numerous in vitro studies. Inflammation triggered by microglia plays an important role in promoting neurodegeneration by inducing the expression of pro-inflammatory factors [102,161,162,163]. In this way, LPS-induced inflammatory neurotoxicity depends on the excessive production of pro-inflammatory factors by microglia [164]. Activation of TLR4 on the cell membrane by LPS activates various signal cascades, including NF-κB via the MyD88–IRAK–TRAF6–TAK1 signaling complex [38,165,166,167]. Upon LPS stimulation, the transcription factor NF-κB plays an important role in the expression of pro-inflammatory genes via its translocation to the nucleus [168] which can trigger a series of inflammatory pathways. 

LPS stimulation of BV-2 microglial cells [169], co-cultures of neurons, astrocytes, and microglia [170], or hippocampal neurons cultures [171] resulted in increased synthesis and release of IL-1β and TNF-α. Besides its pro-inflammatory activity, LPS affected the viability of neurons, leading to highly condensed nuclei and the absence/retraction of neurites [170]. Treatment with LPS activated microglia also in rat basal forebrain mixed neuron–glial cultures. Additionally, the number of choline acetyltransferase-immunopositive neurons were decreased in these cultures treated with LPS [172]. Recently, a study also showed the activation of microglia by LPS, which induced corpus callosum nerve fiber malfunction and fast axonal transport [173]. Microglial response induced by LPS was also associated with the activation of COX-2 and the NOS pathway, resulting in a dramatic increase in prostaglandin E_2_ (PGE_2_) and nitric oxide production [172,174,175,176,177,178,179,180,181,182,183], which contributed to neurotoxicity and cellular dysfunction in neuron-glia cultures. 

In mesencephalic mixed neuron–glia cultures, LPS exposure induced the reduction of TH-positive neurons in the presence of glia. However, LPS treatment did not affect dopaminergic cells when neurons were cultured in the absence of glia [89,184], suggesting that the glial-mediated neuronal damage was induced by LPS. Moreover, the increased release of inflammatory mediators IL-1β and TNF-α induced by LPS was associated with decreased TH-positive cells in primary mesencephalic cultures, which was prevented by using neutralizing antibodies against IL-1β or TNF-α [185]. In contrast, pretreatment with the anti-inflammatory cytokine IL-10 prevented dopaminergic neuron loss induced by LPS in primary ventral mesencephalic cultures due to a reduced production of proinflammatory cytokines and protection against a reduction of neurotrophic factors [186]. 

Finally, LPS treatment reduced the DA reuptake capacity of dopaminergic neurons in the neuron–glia cultures [187], exposing other aspects that might contribute to PD pathology.

Many protein kinases, such as p38 mitogen-activated protein kinases (p38 MAPK) and protein kinase C-δ (PKCδ) have been implicated in the release of inflammatory mediators from glia, resulting in neuronal death [188,189,190]. p38 MAPK mediates LPS-induced neurodegeneration in mesencephalic neuron–glia cultures through the induction of nitric oxide synthase resulting in increased NO production [179]. Another study using U373 cells showed an increased IL-6 production by stimulation with LPS, mediated by the p38/Src kinase inhibitors-dependent pathway [77]. Treatment of primary and BV-2 microglial cultures with LPS resulted in increased activation of phospho-p38 MAPK [178,181,182,183,191,192,193]. In addition, PKCδ was highly upregulated during chronic microglial activation, and a significant increase in PKCδ kinase activity was observed [190], followed by ROS generation, NO production, and proinflammatory cytokine and chemokine release. Proteolytic activation of PKCδ occurred during dopaminergic degeneration and was mediated by caspase-3 [194,195,196]. Silencing of caspase-3 or AIF by small interfering RNAs, exclusively in DA MN9D cells, protected DA cells from LPS-induced death, demonstrating the key role of these molecules in LPS-induced neurotoxicity [96].

Finally, LPS increased the expression levels of β-site APP cleaving enzyme 1 (BACE-1), PS-1, β-APP, and Aβ1-42 in neuron cultures treated with LPS [171]. LPS exposure also contributed synergistically to the negative effects of α-synuclein on progressive dopaminergic degeneration, associated with increased microglial superoxide production [197]. In addition, LPS could also induce conformational changes in α-synuclein protein, which might accelerate the progression of PD [198]. 

It is noteworthy that in vitro investigations are widely used for the evaluation of mechanisms associated with cell homeostasis or dysfunction. Data from cell cultures therefore also contribute to the better understanding of gaps in intracellular signaling, molecular aspects, gene transcription, mRNA translation, and protein synthesis involved in cell physiology. In this context, LPS-induced in vitro models are very relevant to support the elucidation of the pathophysiology of neurodegenerative diseases. 

## 6. Final Considerations and Conclusions

In the context of AD, models that use LPS contribute to the understanding of the intricate relationship between neuroinflammation and the progression of the disease, mainly in regard to Aβ processing and deposition. Besides, activation of TLR4 and of the inflammatory pathways leads to glial reaction and neuronal loss, which contributes to memory impairment and behavioral changes. Importantly, both acute and chronic inflammation seem to play a role in this neurodegenerative disease. 

On the other hand, injection of LPS per se may be used as an animal model of PD, mainly because of the high susceptibility of mesencephalic neurons to this toxin [89,131,199]. In this sense, injection of LPS can contribute to the elucidation of the inflammatory pathways that induce glial activation and the additional causes of neuronal death, dopamine signaling disbalance, α-syn aggregation, and behavioral symptoms. Finally, in regard to ALS and HD, the role of inflammatory processes in these two neurodegenerative diseases needs to be better studied and elucidated. The studies may consider to include the use of the already established models to evaluate the impact of inflammatory challenges in the development of these pathological conditions.

Importantly, the variety of protocols and serotypes of LPS used in the studies may induce a plethora of results. This wide range of outcomes may contribute to the better understanding of the intricate link between neurodegenerative diseases and peripheral and central inflammation.

In conclusion, LPS is an important tool for the evaluation of different parameters associated with inflammatory processes and may be used in studies that aim to investigate the pathophysiological mechanisms of neurodegenerative diseases. However, the serotype, route of administration, doses, and other parameters should be considered when planning experimental protocols because of the varied responses induced by the endotoxin.

## Figures and Tables

**Table 1:** Lipopolysaccharide (LPS) source, species used, dose and route of administration, duration, evaluated parameters of models of central LPS challenges for the elucidation of Alzheimer’s disease (AD).

LPS	Species Used	Dose and Route of Administration	LPS Injection (Duration)	Evaluated Parameters	References
*E. coli* O127:B8 (Sigma-Aldrich)	Charles River CD-VAF rats	10 ng/animal (intracerebroventricular)	Acute	IL-1 in brain regions: cerebellum, cortex, brainstem, diencephalon, or hippocampus	[35]
*E. coli* O55:B5 (Sigma-Aldrich)	Sprague Dawley	1.0 μg/mL (4th ventricle)	Chronic (four weeks)	Spatial working memory Activation of astrocytes and microglia	[19]
*E. coli* O55:B5 (Sigma-Aldrich)	Fisher-344 rats	0.25 μL/h (4th ventricle)	Chronic (28 days)	Long-term depression (LTD) Underlying mechanism of LTD impairment by neuroinflammation	[20]
*S. abortus equi* (Sigma-Aldrich)	Tg2576 APP mice	4 μg/μL or 10 μg/μL (intrahippocampal)	Acute	Amyloid-beta (Aβ) load Microglial and astrocytes activation over time	[46]
*S. abortus equi* (Sigma-Aldrich)	Nontransgenic mice obtained during breeding of our amyloid precursor protein (APP)1 + presenilin (PS)1 transgenic mouse colony	1 μL of 4 μg/μL (intrahippocampal; bilateral)	Acute	Time course of microgliosis Time course of astrogliosis Time course of TLR4 levels Quantification of glial markers (GFAP, CD45) TNF-α and IL-1β levels	[40]
*S. abortus equi* (Sigma-Aldrich)	Tg2576 APP mice	10 μg/μL (intrahippocampal; unilateral)	Acute	Brain amyloid burden Markers of microglial activation (CD45, CR3 or CD11b, CD68, Fcg receptor, and scavenger receptor A)	[47]
*S. typhimurium* (Sigma-Aldrich)	APP1PS1 transgenic mice were transplanted with eGFP-over-expressing bone marrow	4 μg of LPS (4 μg/μL in saline); (intrahippocampal; unilateral)	Acute	Proliferation, expression of markers for activated microglia Aβ removal	[48]
*S. abortus equii* (Sigma-Aldrich)	rTg4510 mice and non-transgenic mice	5 μg/μL (frontal cortex and hippocampus)	Acute	Activation of CD45 and arginase 1 Expression of Ser199/202 and phospho-tau Ser396	[44]
*E. coli* O55:B5 (Sigma-Aldrich)	Sprague Dawley rats	2.5 μg/μL (intrahippocampal; unilateral)	Acute	β-secretase-1 (BACE1) and GFAP levels Amyloidogenic protein expression Golgi preparations of cortical layer III pyramidal neurons	[41]
*S. abortus equi* (Sigma-Aldrich)	TgAPP/PS1 and C57BL/6	4 μg/μL (2-month-old mice) or 2 μg/μL (12-month-old mice) (intrahippocampal)	Acute	Aβ deposits in the hippocampus and cortex Activation of microglia	[45]

**Table 2:** LPS source, species used, dose and route of administration, duration, evaluated parameters of models of systemic LPS challenges for the elucidation of AD.

LPS	Species Used	Dose and Route of Administration	LPS Injection (Duration)	Evaluated Parameters	References
*E. coli* O127:B8 (Sigma-Aldrich)	Charles River CD-VAF rats	1 mg/kg (intraperitoneal)	Acute	Detection of IL-1 by thymocyte stimulation	[35]
*E. coli* O111:B4 (Sigma-Aldrich)	TgN(APP-Sw) 2576	0.25 μg/μL (intravenously)	Acute	Aβ levels in cortex and hippocampus IL-1β levels in cortex and hippocampus	[52]
*E. coli* O55:B5 (Sigma-Aldrich)	3xTg-AD or nontransgenic mice	0.1 mg/mL; 0.5 mg/kg body weight (intraperitoneal)	Chronic (twice a week for six weeks)	Characterization of time course of microglia activation in the brain Microglial activation and tangle pathology	[74]
*E. coli* O55:B5 (Sigma-Aldrich)	ICR mice	250 μg/kg (intraperitoneal)	Acute (daily for three or seven days)	Memory impairment Aβ accumulation in the cortex and hippocampus Expression of amyloidogenic proteins Astrocytes activation	[59]
*E. coli* O111:B4 (Sigma-Aldrich)	3xTgAD mice	0.25 mg/kg (intraperitoneal)	Chronic (twice weekly for four weeks)	Effect of inhibition of soluble TNF signaling on accumulation of 6E10-immunoreactive protein in hippocampus, cortex, and amygdala and amyloid-associated pathology	[58]
*S. typhimurium* (Sigma-Aldrich)	CD-1 mice	3, 30, 300, or 3000 μg/kg (intraperitoneal)	Acute	Transport of Aβ across the blood–brain barrier	[69]
*E. coli* O55:B5 (Sigma-Aldrich)	Wistar	5 mg/kg (intraperitoneal)	Acute	Cognitive functions (amnesic, discriminative, and attentional functions) Anxiety TNF and IL-18 protein levels in frontal cortex, hippocampus, striatum, cerebellum, and hypothalamus	[54]
*S. typhimurium* (Sigma-Aldrich)	CD-1 mice	3 mg/kg (intraperitoneal)	Acute	Aβ transporter across the blood-brain barrier Oxidative stress markers in brain and serum Brain influx of I-albumin IL-1α, IL-1β, IL-6, IL-12, IL-13, MIP-1α, MIP-1β, G-CSF, KC, MCP-1, RANTES, and TNF-α levels in cortex and hippocampus	[67]
*S. typhimurium* (Sigma-Aldrich)	CD-1 mice	3 mg/kg (intraperitoneal)	Acute	Quantification of LRP-1 LRP-1-dependent partitioning between the brain vasculature and parenchyma and peripheral clearance	[70]
*E. coli* O55:B5 (Sigma-Aldrich)	Wistar rats	500 µg/kg/day (intraperitoneal)	For seven consecutive days.	Nitric oxide (NO) production NO synthase (NOS2) Aβ 1-42 cerebral expression Memory	[56]
*E. coli* O8:K27 (Innaxon)	EFAD mice (express human APOE3 or APOE4 and overproduce human Aβ	0.5 mg/kg/week (intraperitoneal)	Chronic (from 4 to 6 months of age)	Cognitive dysfunction Cerebrovascular leakiness Aβ42 levels Cerebral amyloid angiopathy-like deposition IL-10, G-CSF, RANTES, IL-12, IL-17, KC levels	[57]
*E. coli* O111:B4 (Sigma-Aldrich)	APP_SWE_/PS11_∆E9_ Tg and wild-type	0.5 mg/kg (intraperitoneal)	Chronic (Once a week for 13 weeks)	TNF and IL-1β mRNA levels Amyloid pathology in the neocortex CD11b+ cells clustering around Aβ plaques APP, APOE, Clu, and Hexb protein expression in neocortex	[60]
*E. coli* O111:B4 (Sigma-Aldrich)	5XFAD and C57BL/6 mice	0.01 mg/kg, 0.1 mg/kg, 1 mg/kg, 3 mg/kg (intravenously)	Acute	Disruption of blood–brain barrier Delivery of large molecules through the blood–brain barrier Weight loss	[71]

**Table 3:** LPS source, species used, dose and route of administration, duration, evaluated parameters of models of central LPS challenges for the elucidation of Parkinson’s disease (PD).

LPS	Species Used	Dose and Route of Administration	LPS Injection (Duration)	Evaluated Parameters	References
*E. coli* O26:B6 (Sigma-Aldrich)	Wistar	1 mg/mL (2 µL intranigral)	Acute	Dopamine (DA) and DA metabolites Loss of tyrosine hydroxylase (TH)-positive cells TH activity Microglial activation NOS inhibition	[87,88]
*E. coli* O111:B4 (Life Technologies)	Fischer 344	5 or 10 µg in 2 µL (intrastriatal, intrahippocampal or intracortical)	Acute	Loss of TH-positive cells MAP-2-positive cell loss Microglial activation	[89]
*E. coli* O111:B4 (Sigma-Aldrich)	Sprague–Dawley rats	5 µg in 2 µL (intranigral)	Acute	Loss of TH-positive cells Microglial activation Naloxone effects on LPS consequences	[92]
*E. coli* O26:B6 (Sigma-Aldrich)	Wistar	5 µg (intranigral)	Acute	Dopamine and DA metabolites Serotonin and DA metabolites TH activity Loss of TH-positive cells Glial reaction Effects of dexamethasone on LPS consequences	[93]
*E. coli* O111:B4 (Sigma-Aldrich)	Fischer 344	5 ng/h (intranigral)	Chronic (2 weeks)	Loss of TH-positive cells Loss of NeuN-positive cells Microglial activation	[90]
*E. coli* O26:B6 (Sigma-Aldrich)	Wistar	10 µg (intranigral)	Acute	Loss of TH-positive cells Loss of FG-labelled neurons NADPH-d expression iNOS expression	[110]
*E. coli* O55:B5 (Calbiochem)	Wistar	10 µg (supranigral)	Acute	Loss of TH-positive cells Motor evaluation Astrocyte reaction Microglial activation iNOS expression Neurotophin-3 expression	[91]
*E. coli* O111:B4 (Sigma-Aldrich)	Fischer 344	10 µg (intrapallidal)	Acute	Loss of TH-positive cells Microglial activation Ferritin expression Iron levels A-synuclein expression Ubiquitin expression Effect of aging on LPS consequences	[113]
*E. coli* O26:B6 (Sigma-Aldrich)	Wistar	2 mg/mL (intranigral)	Acute	Loss of TH-positive cells Microglial activation TH expression Cytokine mRNA expression iNOS expression Caspase-11 expression Effects of p38 MAPK inhibition in LPS consequences Effects of iNOS blockage on LPS consequences	[109]
*S. minnesota* (Sigma-Aldrich)	Sprague-Dawley	16, 32 or 60 µg (intrastriatal)	Acute	DA and DA metabolites Loss of TH-positive cells Microglial activation Pro-inflammatory cytokine expression Insulin receptor expression Mitochondrial activity Effects of cyclooxygenase-2 (COX-2) inhibition and PPAR-c agonist on LPS consequences	[112]
*S. minnesota* (Sigma-Aldrich)	Sprague-Dawley	16 µg (intrastriatal)	Acute	UCP2 expression mitoNEET expression Effects of PPAR-c agonist on LPS consequences	[107]
*S. minnesota* (Sigma-Aldrich)	C57BL/6	5, 7.5, or 10 μg (intrastriatal)	Acute	Loss of TH-positive cells Motor evaluation NOS expression Effects of NOS inhibition in LPS consequences Effects of iNOS knockout on LPS consequences	[111]
*S. minnesota* (Sigma-Aldrich)	Wistar	2.5 µg/µL (intrastriatal)	Acute	DA Nigrostriatal system evaluation a-synuclein expression Ubiquitin expression Motor evaluation Microglial activation iNOS expression Mitochondrial activity	[108]
*E. coli* O111:B4 (Calbiochem)	Fischer 344	5 µg (intranigral)	Acute	Loss of TH-positive cells Microglial activation Effects of IκB Kinase-β inhibition on LPS consequences	[187]
*E. coli* O26:B6 (Sigma-Aldrich)	ABH-Biozzi	0.5 mg/kg	Acute	NFκB mRNA expression Cell death evaluation	[95]
*E. coli* (Sigma-Aldrich)	C57BL/6	10 μg (intrastriatal)	Acute	Motor evaluation DA neuron loss DA and DA metabolites Microglial activation Iron concentration Effects of desferrioxamine on the LPS consequences	[114]
*E. coli* O55:B5 (Sigma-Aldrich)	Fischer 344	0.25 μg/h (intracerebroventricular)	Chronically (21 or 56 days)	Cytokine protein levels Cytokine mRNA expression Loss of TH-positive cells MHC II-IR microglial density Effects of aging on LPS consequences	[97]
*E. coli* O111:B4 (Sigma-Aldrich)	Sprague-Dawley	5 μg/5 μL (intranigral)	Acute	Astrocyte reaction Microglial activation NFκB transcription Cytokine transcription NOX2 activation NADPH-Oxidase Activity Reactive oxygen species (ROS) production Lipid peroxidation iNOS and NO expression. DA and DA metabolites Effects of NADPH-oxidase inhibition on LPS consequences	[99]
*E. coli* (Sigma-Aldrich)	SD rats	5 mg/mL (intrastriatal)	Acute	Motor evaluation Glial activation Oxidative stress Apoptosis	[103]
*S. minnesota* (Sigma-Aldrich)	Sprague-Dawley	32 μg (intrastriatal)	Acute	Mitochondrial activity and structure Oxidative stress Loss of TH-positive cells	[106]
*E. coli* O55:B5 (Sigma-Aldrich)	Wistar	5 μg/2 μL (intranigral)	Acute	Fever and Sickness Microglial Activation and phagocytic activity Astrocyte Activation Oxidative Stress Cytokine levels Leukocyte brain Infiltration	[98]
*E. coli* O111:B4 (Enzo Life Science)	*LRRK2* KO C57BL/6 and wild-type	5 mg/mL (intrastriatal)	Acute	Microglial activation Role of LRRK2 on LPS consequences	[121]
*E. coli* (Sigma-Aldrich)	DJ-1 KO C57BL/6 and wild-type	1 μg/μL (intranigral)	Acute	Dopaninergic normal loss sICAM-1, IFN-γ, IL-1β, IL-1Ra, IL-16, IL-17, and I-TAC expression Role of DJ-1 on LPS consequences	[124]

**Table 4:** LPS source, species used, dose and route of administration, duration, evaluated parameters of models of systemic LPS challenges for the elucidation of PD.

LPS	Species Used	Dose and Route of Administration	LPS Injection (Duration)	Evaluated Parameters	References
*E. coli* O55:B5 (Sigma-Aldrich)	C57BL/6	1 mg/kg (intraperitoneal)	Acute (single dose)	Ultrastructural Alterations in SN NOS Activity NOS and TNF expression Apoptotic Pathways	[132]
*E. coli* O111:B4 (Calbiochem)	C57BL/6, TNFR1/R2^−/−^ KO, TNFR1/R2^+/+^ WT	5 mg/kg (intraperitoneal)	Acute (single dose)	TNFα level Loss of TH-positive cells Effects of TNFR knock-out on LPS consequences	[127]
*E. coli* O111:B4 (Sigma-Aldrich)	C57BL/6	5 mg/kg (intraperitoneal)	Weekly injected with five doses of LPS Monthly injected with two to five doses of LPS	Motor evaluation Loss of TH-positive cells α-synuclein accumulation Microglial activation Sex differences in LPS consequences	[129]
*E. coli* O111:B4 (Sigma-Aldrich)	B6C3F1 WT and transgenic mice for mutant α-synuclein	3 × 10^6^ EU/kg (intraperitoneal)	Acute (single injection)	Nigral TH-positive cells evaluation α-synuclein aggregation Cytokine levels Microglial activation Differences in acute and chronic neuroinflammation Effects iNOS inhibition of iNOS inhibition and NADPH oxidase blockage on LPS consequences	[61]
*E. coli* O111:B4	C57BL/6	0.2 mg/kg (intraperitoneal)	Acute (single injection)	Cytokine expression. TH-positive cells evaluation Microglial activation iNOS mRNA expression NF-κB mRNA expression. gp91phox level Oxidative stress Effects of HCT1026 on LPS consequences	[134]
*E. coli* O55:B5 (Sigma-Aldrich)	129/SvEv and α-syn gene-ablated mice	1 mg/kg (intraperitoneal)	Acute (single dose)	Blood–brain barrier integrity	[130]
*E. coli* O111:B4 (Calbiochem)	B6.129S6-Cybbtm1Din (NOX2−/−) and C57BL/6 000664 (NOX2+/+)	5 mg/kg (intraperitoneal)	Acute (single injection)	NOX2 expression ROS production Microglial activation Effects of oxidases inhibition on LPS consequences	[133]
*E. coli* (Sigma-Aldrich)	C57BL/6	5 mg/kg (intraperitoneal)	Acute (single injection)	TH-positive cells evaluation α-syn aggregation and levels Microglial activation Autophagic activity	[131]
*E. coli* O111:B4 (Sigma-Aldrich)	C57BL/6 and PKCδ KO mice	5 mg/kg (intraperitoneal)	Acute (single injection)	Motor evaluation Cytokine release and expression. Effects of PKCδ KO on LPS consequences	[190]
*S. abortus equi* (Enzo Life Sciences)	C57BL/6	1 μg/g (intraperitoneal)		Motor evaluation TH-positive cells evaluation DA and DA metabolites Microglial and astrocytic activation Cytokine levels and expression	[128]

**Table 5:** LPS source, species used, dose and route of administration, duration, evaluated parameters of models of systemic LPS challenges for the elucidation of amyotrophic lateral sclerosis (ALS).

LPS	Species Used	Dose and Route of Administration	LPS Injection (Duration)	Evaluated Parameters	References
*E. coli* O55:B5 (Calbiochem)	C57BL/6 EP4 floxed mice	5 mg/kg (intraperitoneal)	Acute	Quantification of COX-2, iNOS, TNF-α, IL-6, and IL-1β mRNA levels in hippocampus	[175]
*E. coli* O55:B5 (Sigma-Aldrich)	G93A-SOD1 C/EBPδ^(−/−)^ mice	200 μg/animal (intraperitoneal) 1 μg/μL (intraperitoneal)	Acute 2, 8, 16, 24, and 48 h	C/EBPδ expression in mouse brain Quantification of NOS-2, COX-2, TNF-α, IL-1β, and IL-6 mRNA TNF-α, IL-1β and IL-6 serum levels	[142]
*E. coli* O55:B5 (Sigma-Aldrich)	TDP-43^A315T^ and C57BL/6 mice	1 mg/kg of body weight (intraperitoneal)	Chronic (Once a week for two months)	TDP-43 accumulation in the cytoplasm of spinal motor neurons TDP-43 aggregation	[145]

**Table 6:** LPS source, species used, dose and route of administration, duration, evaluated parameters of models of systemic LPS challenges for the elucidation of Huntington’s disease (HD).

LPS	Species Used	Dose and Route of Administration	LPS Injection (Duration)	Evaluated Parameters	References
*E. coli* (Sigma-Aldrich)	Transgenic YAC128 and wild type	1 mg/kg (intraperitoneal)	Chronic (Once a week for four months)	Microglial activation Neurovascular integrity Blood brain barrier integrity	[159]
*E. coli* O111:B4 (Sigma-Aldrich)	Transgenic R6/2 and wild type	2 mg/kg (intraperitoneal)	Acute	NF-κB activation Inflammatory evaluation Motor evaluation	[62]
*E. coli* O127:B8 (Sigma-Aldrich)	Transgenic R6/2 and wild type	0.3 mg/kg (intraperitoneal)	Acute	TNF gene expression. IL-6 gene expression Sex-dependent effects of LPS injection	[160]
*E. coli* O111:B4 (Sigma-Aldrich)	Transgenic R6/2 and wild type	2 μg/animal (intraperitoneal)	Chronic (Once a week for seven weeks)	Splenic immune cells evaluation T-cell activity Motor evaluation	[65]

## References

[1] Ransohoff R.M. (2016). How neuroinflammation contributes to neurodegeneration. Science.

[2] Boonen B., Alpizar Y.A., Sanchez A., Lopez-Requena A., Voets T., Talavera K. (2018). Differential effects of lipopolysaccharide on mouse sensory TRP channels. Cell Calcium.

[3] Alpizar Y.A., Boonen B., Sanchez A., Jung C., Lopez-Requena A., Naert R., Steelant B., Luyts K., Plata C., De Vooght V. (2017). TRPV4 activation triggers protective responses to bacterial lipopolysaccharides in airway epithelial cells. Nat. Commun..

[4] Meseguer V., Alpizar Y.A., Luis E., Tajada S., Denlinger B., Fajardo O., Manenschijn J.A., Fernandez-Pena C., Talavera A., Kichko T. (2014). TRPA1 channels mediate acute neurogenic inflammation and pain produced by bacterial endotoxins. Nat. Commun..

[5] Fitzgerald K.A., McWhirter S.M., Faia K.L., Rowe D.C., Latz E., Golenbock D.T., Coyle A.J., Liao S.M., Maniatis T. (2003). IKKepsilon and TBK1 are essential components of the IRF3 signaling pathway. Nat. Immunol..

[6] Ruckdeschel K., Pfaffinger G., Haase R., Sing A., Weighardt H., Hacker G., Holzmann B., Heesemann J. (2004). Signaling of apoptosis through TLRs critically involves toll/IL-1 receptor domain-containing adapter inducing IFN-beta, but not MyD88, in bacteria-infected murine macrophages. J. Immunol..

[7] Zughaier S.M., Zimmer S.M., Datta A., Carlson R.W., Stephens D.S. (2005). Differential induction of the toll-like receptor 4-MyD88-dependent and -independent signaling pathways by endotoxins. Infect. Immun..

[8] Gray P., Dagvadorj J., Michelsen K.S., Brikos C., Rentsendorj A., Town T., Crother T.R., Arditi M. (2011). Myeloid differentiation factor-2 interacts with Lyn kinase and is tyrosine phosphorylated following lipopolysaccharide-induced activation of the TLR4 signaling pathway. J. Immunol..

[9] Park B.S., Lee J.O. (2013). Recognition of lipopolysaccharide pattern by TLR4 complexes. Exp. Mol. Med..

[10] Acosta C., Davies A. (2008). Bacterial lipopolysaccharide regulates nociceptin expression in sensory neurons. J. Neurosci. Res..

[11] Leow-Dyke S., Allen C., Denes A., Nilsson O., Maysami S., Bowie A.G., Rothwell N.J., Pinteaux E. (2012). Neuronal Toll-like receptor 4 signaling induces brain endothelial activation and neutrophil transmigration in vitro. J. Neuroinflamm..

[12] Chistyakov D.V., Azbukina N.V., Lopachev A.V., Kulichenkova K.N., Astakhova A.A., Sergeeva M.G. (2018). Rosiglitazone as a Modulator of TLR4 and TLR3 Signaling Pathways in Rat Primary Neurons and Astrocytes. Int. J. Mol. Sci..

[13] Rolls A., Shechter R., London A., Ziv Y., Ronen A., Levy R., Schwartz M. (2007). Toll-like receptors modulate adult hippocampal neurogenesis. Nat. Cell Biol..

[14] Jahn H. (2013). Memory loss in Alzheimer’s disease. Dialogues Clin. Neurosci..

[15] Nelson P.T., Alafuzoff I., Bigio E.H., Bouras C., Braak H., Cairns N.J., Castellani R.J., Crain B.J., Davies P., Del Tredici K. (2012). Correlation of Alzheimer disease neuropathologic changes with cognitive status: A review of the literature. J. Neuropathol. Exp. Neurol..

[16] Small S.A., Schobel S.A., Buxton R.B., Witter M.P., Barnes C.A. (2011). A pathophysiological framework of hippocampal dysfunction in ageing and disease. Nat. Rev. Neurosci..

[17] Braak H., Braak E. (1991). Neuropathological stageing of Alzheimer-related changes. Acta Neuropathol..

[18] Ittner L.M., Gotz J. (2011). Amyloid-beta and tau--a toxic pas de deux in Alzheimer’s disease. Nat. Rev. Neurosci..

[19] Hauss-Wegrzyniak B., Dobrzanski P., Stoehr J.D., Wenk G.L. (1998). Chronic neuroinflammation in rats reproduces components of the neurobiology of Alzheimer’s disease. Brain Res..

[20] Min S.S., Quan H.Y., Ma J., Lee K.H., Back S.K., Na H.S., Han S.H., Yee J.Y., Kim C., Han J.S. (2009). Impairment of long-term depression induced by chronic brain inflammation in rats. Biochem. Biophys. Res. Commun..

[21] Wee Yong V. (2010). Inflammation in neurological disorders: A help or a hindrance?. Neurosci. Rev. J. Bringing Neurobiol. Neurol. Psychiatry.

[22] Heppner F.L., Ransohoff R.M., Becher B. (2015). Immune attack: The role of inflammation in Alzheimer disease. Nat. Rev. Neurosci..

[23] Salter M.W., Stevens B. (2017). Microglia emerge as central players in brain disease. Nat. Med..

[24] Bauer J., Strauss S., Schreiter-Gasser U., Ganter U., Schlegel P., Witt I., Yolk B., Berger M. (1991). Interleukin-6 and alpha-2-macroglobulin indicate an acute-phase state in Alzheimer’s disease cortices. FEBS Lett..

[25] Mrak R.E., Sheng J.G., Griffin W.S. (1995). Glial cytokines in Alzheimer’s disease: Review and pathogenic implications. Hum. Pathol..

[26] Wyss-Coray T., Rogers J. (2012). Inflammation in Alzheimer disease—A brief review of the basic science and clinical literature. Cold Spring Harb. Perspect. Med..

[27] Miklossy J. (2008). Chronic inflammation and amyloidogenesis in Alzheimer’s disease—Role of Spirochetes. J. Alzheimer’s Dis. JAD.

[28] Sheng J.G., Bora S.H., Xu G., Borchelt D.R., Price D.L., Koliatsos V.E. (2003). Lipopolysaccharide-induced-neuroinflammation increases intracellular accumulation of amyloid precursor protein and amyloid beta peptide in APPswe transgenic mice. Neurobiol. Dis..

[29] Zhan X., Stamova B., Jin L.W., DeCarli C., Phinney B., Sharp F.R. (2016). Gram-negative bacterial molecules associate with Alzheimer disease pathology. Neurology.

[30] Zhao Y., Cong L., Jaber V., Lukiw W.J. (2017). Microbiome-Derived Lipopolysaccharide Enriched in the Perinuclear Region of Alzheimer’s Disease Brain. Front. Immunol..

[31] Zhao Y., Jaber V., Lukiw W.J. (2017). Secretory Products of the Human GI Tract Microbiome and Their Potential Impact on Alzheimer’s Disease (AD): Detection of Lipopolysaccharide (LPS) in AD Hippocampus. Front. Cell. Infect. Microbiol..

[32] Zhao Y., Lukiw W.J. (2018). Bacteroidetes Neurotoxins and Inflammatory Neurodegeneration. Mol. Neurobiol..

[33] Zhan X., Stamova B., Sharp F.R. (2018). Lipopolysaccharide Associates with Amyloid Plaques, Neurons and Oligodendrocytes in Alzheimer’s Disease Brain: A Review. Front. Aging Neurosci..

[34] Zakaria R., Wan Yaacob W.M., Othman Z., Long I., Ahmad A.H., Al-Rahbi B. (2017). Lipopolysaccharide-induced memory impairment in rats: A model of Alzheimer’s disease. Physiol. Res..

[35] Quan N., Sundar S.K., Weiss J.M. (1994). Induction of interleukin-1 in various brain regions after peripheral and central injections of lipopolysaccharide. J. Neuroimmunol..

[36] Minghetti L., Ajmone-Cat M.A., De Berardinis M.A., De Simone R. (2005). Microglial activation in chronic neurodegenerative diseases: Roles of apoptotic neurons and chronic stimulation. Brain Res. Brain Res. Rev..

[37] Lucin K.M., Wyss-Coray T. (2009). Immune activation in brain aging and neurodegeneration: Too much or too little?. Neuron.

[38] Fiebich B.L., Batista C.R.A., Saliba S.W., Yousif N.M., de Oliveira A.C.P. (2018). Role of Microglia TLRs in Neurodegeneration. Front. Cell. Neurosci..

[39] Ophir G., Meilin S., Efrati M., Chapman J., Karussis D., Roses A., Michaelson D.M. (2003). Human apoE3 but not apoE4 rescues impaired astrocyte activation in apoE null mice. Neurobiol. Dis..

[40] Herber D.L., Maloney J.L., Roth L.M., Freeman M.J., Morgan D., Gordon M.N. (2006). Diverse microglial responses after intrahippocampal administration of lipopolysaccharide. Glia.

[41] Deng X., Li M., Ai W., He L., Lu D., Patrylo P.R., Cai H., Luo X., Li Z., Yan X. (2014). Lipolysaccharide-Induced Neuroinflammation Is Associated with Alzheimer-Like Amyloidogenic Axonal Pathology and Dendritic Degeneration in Rats. Adv. Alzheimer’s Dis..

[42] Philippens I.H., Ormel P.R., Baarends G., Johansson M., Remarque E.J., Doverskog M. (2017). Acceleration of Amyloidosis by Inflammation in the Amyloid-Beta Marmoset Monkey Model of Alzheimer’s Disease. J. Alzheimer’s Dis. JAD.

[43] Mrdjen D., Pavlovic A., Hartmann F.J., Schreiner B., Utz S.G., Leung B.P., Lelios I., Heppner F.L., Kipnis J., Merkler D. (2018). High-Dimensional Single-Cell Mapping of Central Nervous System Immune Cells Reveals Distinct Myeloid Subsets in Health, Aging, and Disease. Immunity.

[44] Lee D.C., Rizer J., Selenica M.L., Reid P., Kraft C., Johnson A., Blair L., Gordon M.N., Dickey C.A., Morgan D. (2010). LPS-induced inflammation exacerbates phospho-tau pathology in rTg4510 mice. J. Neuroinflamm..

[45] Go M., Kou J., Lim J.E., Yang J., Fukuchi K.I. (2016). Microglial response to LPS increases in wild-type mice during aging but diminishes in an Alzheimer’s mouse model: Implication of TLR4 signaling in disease progression. Biochem. Biophys. Res. Commun..

[46] Herber D.L., Roth L.M., Wilson D., Wilson N., Mason J.E., Morgan D., Gordon M.N. (2004). Time-dependent reduction in Abeta levels after intracranial LPS administration in APP transgenic mice. Exp. Neurol..

[47] Herber D.L., Mercer M., Roth L.M., Symmonds K., Maloney J., Wilson N., Freeman M.J., Morgan D., Gordon M.N. (2007). Microglial activation is required for Abeta clearance after intracranial injection of lipopolysaccharide in APP transgenic mice. J. Neuroimmune Pharmacol. Off. J. Soc. NeuroImmune Pharmacol..

[48] Malm T.M., Magga J., Kuh G.F., Vatanen T., Koistinaho M., Koistinaho J. (2008). Minocycline reduces engraftment and activation of bone marrow-derived cells but sustains their phagocytic activity in a mouse model of Alzheimer’s disease. Glia.

[49] Perry V.H. (2004). The influence of systemic inflammation on inflammation in the brain: Implications for chronic neurodegenerative disease. Brain Behav. Immun..

[50] Lee J., Chan S.L., Mattson M.P. (2002). Adverse effect of a presenilin-1 mutation in microglia results in enhanced nitric oxide and inflammatory cytokine responses to immune challenge in the brain. Neuromol. Med..

[51] Brugg B., Dubreuil Y.L., Huber G., Wollman E.E., Delhaye-Bouchaud N., Mariani J. (1995). Inflammatory processes induce beta-amyloid precursor protein changes in mouse brain. Proc. Natl. Acad. Sci. USA.

[52] Sly L.M., Krzesicki R.F., Brashler J.R., Buhl A.E., McKinley D.D., Carter D.B., Chin J.E. (2001). Endogenous brain cytokine mRNA and inflammatory responses to lipopolysaccharide are elevated in the Tg2576 transgenic mouse model of Alzheimer’s disease. Brain Res. Bull..

[53] Wang L.M., Wu Q., Kirk R.A., Horn K.P., Ebada Salem A.H., Hoffman J.M., Yap J.T., Sonnen J.A., Towner R.A., Bozza F.A. (2018). Lipopolysaccharide endotoxemia induces amyloid-beta and p-tau formation in the rat brain. Am. J. Nucl. Med. Mol. Imaging.

[54] Bossu P., Cutuli D., Palladino I., Caporali P., Angelucci F., Laricchiuta D., Gelfo F., De Bartolo P., Caltagirone C., Petrosini L. (2012). A single intraperitoneal injection of endotoxin in rats induces long-lasting modifications in behavior and brain protein levels of TNF-alpha and IL-18. J. Neuroinflamm..

[55] Katafuchi T., Ifuku M., Mawatari S., Noda M., Miake K., Sugiyama M., Fujino T. (2012). Effects of plasmalogens on systemic lipopolysaccharide-induced glial activation and beta-amyloid accumulation in adult mice. Ann. N. Y. Acad. Sci..

[56] Behairi N., Belkhelfa M., Rafa H., Labsi M., Deghbar N., Bouzid N., Mesbah-Amroun H., Touil-Boukoffa C. (2016). All-trans retinoic acid (ATRA) prevents lipopolysaccharide-induced neuroinflammation, amyloidogenesis and memory impairment in aged rats. J. Neuroimmunol..

[57] Marottoli F.M., Katsumata Y., Koster K.P., Thomas R., Fardo D.W., Tai L.M. (2017). Peripheral Inflammation, Apolipoprotein E4, and Amyloid-beta Interact to Induce Cognitive and Cerebrovascular Dysfunction. ASN Neuro.

[58] McAlpine F.E., Lee J.K., Harms A.S., Ruhn K.A., Blurton-Jones M., Hong J., Das P., Golde T.E., LaFerla F.M., Oddo S. (2009). Inhibition of soluble TNF signaling in a mouse model of Alzheimer’s disease prevents pre-plaque amyloid-associated neuropathology. Neurobiol. Dis..

[59] Lee J.W., Lee Y.K., Yuk D.Y., Choi D.Y., Ban S.B., Oh K.W., Hong J.T. (2008). Neuro-inflammation induced by lipopolysaccharide causes cognitive impairment through enhancement of beta-amyloid generation. J. Neuroinflamm..

[60] Thygesen C., Ilkjaer L., Kempf S.J., Hemdrup A.L., von Linstow C.U., Babcock A.A., Darvesh S., Larsen M.R., Finsen B. (2018). Diverse Protein Profiles in CNS Myeloid Cells and CNS Tissue From Lipopolysaccharide- and Vehicle-Injected APPSWE/PS1DeltaE9 Transgenic Mice Implicate Cathepsin Z in Alzheimer’s Disease. Front. Cell. Neurosci..

[61] Gao H.M., Zhang F., Zhou H., Kam W., Wilson B., Hong J.S. (2011). Neuroinflammation and alpha-synuclein dysfunction potentiate each other, driving chronic progression of neurodegeneration in a mouse model of Parkinson’s disease. Environ. Health Perspect..

[62] Hsiao H.Y., Chen Y.C., Chen H.M., Tu P.H., Chern Y. (2013). A critical role of astrocyte-mediated nuclear factor-kappaB-dependent inflammation in Huntington’s disease. Hum. Mol. Genet..

[63] Turner R.C., Naser Z.J., Lucke-Wold B.P., Logsdon A.F., Vangilder R.L., Matsumoto R.R., Huber J.D., Rosen C.L. (2017). Single low-dose lipopolysaccharide preconditioning: Neuroprotective against axonal injury and modulates glial cells. Neuroimmunol. Neuroinflamm..

[64] Wang D., Liu Y., Zhao Y.R., Zhou J.L. (2016). Low dose of lipopolysaccharide pretreatment can alleviate the inflammatory response in wound infection mouse model. Chin. J. Traumatol. = Zhonghua Chuang Shang Za Zhi.

[65] Lee S.W., Park H.J., Im W., Kim M., Hong S. (2018). Repeated immune activation with low-dose lipopolysaccharide attenuates the severity of Huntington’s disease in R6/2 transgenic mice. Anim. Cells Syst..

[66] Cockerill I., Oliver J.A., Xu H., Fu B.M., Zhu D. (2018). Blood-Brain Barrier Integrity and Clearance of Amyloid-beta from the BBB. Adv. Exp. Med. Biol..

[67] Erickson M.A., Hansen K., Banks W.A. (2012). Inflammation-induced dysfunction of the low-density lipoprotein receptor-related protein-1 at the blood-brain barrier: Protection by the antioxidant N-acetylcysteine. Brain Behav. Immun..

[68] Kanekiyo T., Bu G. (2014). The low-density lipoprotein receptor-related protein 1 and amyloid-beta clearance in Alzheimer’s disease. Front. Aging Neurosci..

[69] Jaeger L.B., Dohgu S., Sultana R., Lynch J.L., Owen J.B., Erickson M.A., Shah G.N., Price T.O., Fleegal-Demotta M.A., Butterfield D.A. (2009). Lipopolysaccharide alters the blood-brain barrier transport of amyloid beta protein: A mechanism for inflammation in the progression of Alzheimer’s disease. Brain Behav. Immun..

[70] Erickson M.A., Hartvigson P.E., Morofuji Y., Owen J.B., Butterfield D.A., Banks W.A. (2012). Lipopolysaccharide impairs amyloid beta efflux from brain: Altered vascular sequestration, cerebrospinal fluid reabsorption, peripheral clearance and transporter function at the blood-brain barrier. J. Neuroinflamm..

[71] Barton S.M., Janve V.A., McClure R., Anderson A., Matsubara J.A., Gore J.C., Pham W. (2018). Lipopolysaccharide Induced Opening of the Blood Brain Barrier on Aging 5XFAD Mouse Model. J. Alzheimer’s Dis. JAD.

[72] Ma L., Zhang H., Liu N., Wang P.Q., Guo W.Z., Fu Q., Jiao L.B., Ma Y.Q., Mi W.D. (2016). TSPO ligand PK11195 alleviates neuroinflammation and beta-amyloid generation induced by systemic LPS administration. Brain Res. Bull..

[73] Roe A.D., Staup M.A., Serrats J., Sawchenko P.E., Rissman R.A. (2011). Lipopolysaccharide-induced tau phosphorylation and kinase activity--modulation, but not mediation, by corticotropin-releasing factor receptors. Eur. J. Neurosci..

[74] Kitazawa M., Oddo S., Yamasaki T.R., Green K.N., LaFerla F.M. (2005). Lipopolysaccharide-induced inflammation exacerbates tau pathology by a cyclin-dependent kinase 5-mediated pathway in a transgenic model of Alzheimer’s disease. J. Neurosci. Off. J. Soc. Neurosci..

[75] Maher A., El-Sayed N.S., Breitinger H.G., Gad M.Z. (2014). Overexpression of NMDAR2B in an inflammatory model of Alzheimer’s disease: Modulation by NOS inhibitors. Brain Res. Bull..

[76] Lykhmus O., Voytenko L., Koval L., Mykhalskiy S., Kholin V., Peschana K., Zouridakis M., Tzartos S., Komisarenko S., Skok M. (2015). alpha7 Nicotinic acetylcholine receptor-specific antibody induces inflammation and amyloid beta42 accumulation in the mouse brain to impair memory. PLoS ONE.

[77] Lykhmus O., Mishra N., Koval L., Kalashnyk O., Gergalova G., Uspenska K., Komisarenko S., Soreq H., Skok M. (2016). Molecular Mechanisms Regulating LPS-Induced Inflammation in the Brain. Front. Mol. Neurosci..

[78] Poewe W., Seppi K., Tanner C.M., Halliday G.M., Brundin P., Volkmann J., Schrag A.E., Lang A.E. (2017). Parkinson disease. Nat. Rev. Dis. Prim..

[79] Javoy-Agid F., Agid Y. (1980). Is the mesocortical dopaminergic system involved in Parkinson disease?. Neurology.

[80] Bugiani O., Perdelli F., Salvarani S., Leonardi A., Mancardi G.L. (1980). Loss of striatal neurons in Parkinson’s disease: A cytometric study. Eur. Neurol..

[81] Parkinson J. (2002). An essay on the shaking palsy. J. Neuropsychiatry Clin. Neurosci..

[82] Dauer W., Przedborski S. (2003). Parkinson’s disease: Mechanisms and models. Neuron.

[83] Cookson M.R. (2009). alpha-Synuclein and neuronal cell death. Mol. Neurodegener..

[84] Zhang W., Wang T., Pei Z., Miller D.S., Wu X., Block M.L., Wilson B., Zhang W., Zhou Y., Hong J.S. (2005). Aggregated alpha-synuclein activates microglia: A process leading to disease progression in Parkinson’s disease. FASEB J. Off. Publ. Fed. Am. Soc. Exp. Biol..

[85] McGeer P.L., Itagaki S., Boyes B.E., McGeer E.G. (1988). Reactive microglia are positive for HLA-DR in the substantia nigra of Parkinson’s and Alzheimer’s disease brains. Neurology.

[86] Yang W., Yu S. (2017). Synucleinopathies: Common features and hippocampal manifestations. Cell. Mol. Life Sci..

[87] Castano A., Herrera A.J., Cano J., Machado A. (1998). Lipopolysaccharide intranigral injection induces inflammatory reaction and damage in nigrostriatal dopaminergic system. J. Neurochem..

[88] Herrera A.J., Castano A., Venero J.L., Cano J., Machado A. (2000). The single intranigral injection of LPS as a new model for studying the selective effects of inflammatory reactions on dopaminergic system. Neurobiol. Dis..

[89] Kim W.G., Mohney R.P., Wilson B., Jeohn G.H., Liu B., Hong J.S. (2000). Regional difference in susceptibility to lipopolysaccharide-induced neurotoxicity in the rat brain: Role of microglia. J. Neurosci. Off. J. Soc. Neurosci..

[90] Gao H.M., Jiang J., Wilson B., Zhang W., Hong J.S., Liu B. (2002). Microglial activation-mediated delayed and progressive degeneration of rat nigral dopaminergic neurons: Relevance to Parkinson’s disease. J. Neurochem..

[91] Iravani M.M., Leung C.C., Sadeghian M., Haddon C.O., Rose S., Jenner P. (2005). The acute and the long-term effects of nigral lipopolysaccharide administration on dopaminergic dysfunction and glial cell activation. Eur. J. Neurosci..

[92] Lu X., Bing G., Hagg T. (2000). Naloxone prevents microglia-induced degeneration of dopaminergic substantia nigra neurons in adult rats. Neuroscience.

[93] Castano A., Herrera A.J., Cano J., Machado A. (2002). The degenerative effect of a single intranigral injection of LPS on the dopaminergic system is prevented by dexamethasone, and not mimicked by rh-TNF-alpha, IL-1beta and IFN-gamma. J. Neurochem..

[94] Mangano E.N., Hayley S. (2009). Inflammatory priming of the substantia nigra influences the impact of later paraquat exposure: Neuroimmune sensitization of neurodegeneration. Neurobiol. Aging.

[95] Couch Y., Alvarez-Erviti L., Sibson N.R., Wood M.J., Anthony D.C. (2011). The acute inflammatory response to intranigral alpha-synuclein differs significantly from intranigral lipopolysaccharide and is exacerbated by peripheral inflammation. J. Neuroinflamm..

[96] Burguillos M.A., Hajji N., Englund E., Persson A., Cenci A.M., Machado A., Cano J., Joseph B., Venero J.L. (2011). Apoptosis-inducing factor mediates dopaminergic cell death in response to LPS-induced inflammatory stimulus: Evidence in Parkinson’s disease patients. Neurobiol. Dis..

[97] Bardou I., Kaercher R.M., Brothers H.M., Hopp S.C., Royer S., Wenk G.L. (2014). Age and duration of inflammatory environment differentially affect the neuroimmune response and catecholaminergic neurons in the midbrain and brainstem. Neurobiol. Aging.

[98] Flores-Martinez Y.M., Fernandez-Parrilla M.A., Ayala-Davila J., Reyes-Corona D., Blanco-Alvarez V.M., Soto-Rojas L.O., Luna-Herrera C., Gonzalez-Barrios J.A., Leon-Chavez B.A., Gutierrez-Castillo M.E. (2018). Acute Neuroinflammatory Response in the Substantia Nigra Pars Compacta of Rats after a Local Injection of Lipopolysaccharide. J. Immunol. Res..

[99] Sharma N., Kapoor M., Nehru B. (2016). Apocyanin, NADPH oxidase inhibitor prevents lipopolysaccharide induced alpha-synuclein aggregation and ameliorates motor function deficits in rats: Possible role of biochemical and inflammatory alterations. Behav. Brain Res..

[100] Sharma N., Nehru B. (2016). Apocyanin, a Microglial NADPH Oxidase Inhibitor Prevents Dopaminergic Neuronal Degeneration in Lipopolysaccharide-Induced Parkinson’s Disease Model. Mol. Neurobiol..

[101] Sharma N., Sharma S., Nehru B. (2017). Curcumin protects dopaminergic neurons against inflammation-mediated damage and improves motor dysfunction induced by single intranigral lipopolysaccharide injection. Inflammopharmacology.

[102] Gu C., Hu Q., Wu J., Mu C., Ren H., Liu C.F., Wang G. (2018). P7C3 Inhibits LPS-Induced Microglial Activation to Protect Dopaminergic Neurons Against Inflammatory Factor-Induced Cell Death in vitro and in vivo. Front. Cell. Neurosci..

[103] Xu W., Zheng D., Liu Y., Li J., Yang L., Shang X. (2017). Glaucocalyxin B Alleviates Lipopolysaccharide-Induced Parkinson’s Disease by Inhibiting TLR/NF-kappaB and Activating Nrf2/HO-1 Pathway. Cell. Physiol. Biochem. Int. J. Exp. Cell. Physiol. Biochem. Pharmacol..

[104] Moon H.E., Paek S.H. (2015). Mitochondrial Dysfunction in Parkinson’s Disease. Exp. Neurobiol..

[105] Park J.S., Davis R.L., Sue C.M. (2018). Mitochondrial Dysfunction in Parkinson’s Disease: New Mechanistic Insights and Therapeutic Perspectives. Curr. Neurol. Neurosci. Rep..

[106] Hunter R., Ojha U., Bhurtel S., Bing G., Choi D.Y. (2017). Lipopolysaccharide-induced functional and structural injury of the mitochondria in the nigrostriatal pathway. Neurosci. Res..

[107] Hunter R.L., Choi D.Y., Ross S.A., Bing G. (2008). Protective properties afforded by pioglitazone against intrastriatal LPS in Sprague-Dawley rats. Neurosci. Lett..

[108] Choi D.Y., Liu M., Hunter R.L., Cass W.A., Pandya J.D., Sullivan P.G., Shin E.J., Kim H.C., Gash D.M., Bing G. (2009). Striatal neuroinflammation promotes Parkinsonism in rats. PLoS ONE.

[109] Ruano D., Revilla E., Gavilan M.P., Vizuete M.L., Pintado C., Vitorica J., Castano A. (2006). Role of p38 and inducible nitric oxide synthase in the in vivo dopaminergic cells’ degeneration induced by inflammatory processes after lipopolysaccharide injection. Neuroscience.

[110] Iravani M.M., Kashefi K., Mander P., Rose S., Jenner P. (2002). Involvement of inducible nitric oxide synthase in inflammation-induced dopaminergic neurodegeneration. Neuroscience.

[111] Hunter R.L., Cheng B., Choi D.Y., Liu M., Liu S., Cass W.A., Bing G. (2009). Intrastriatal lipopolysaccharide injection induces parkinsonism in C57/B6 mice. J. Neurosci. Res..

[112] Hunter R.L., Dragicevic N., Seifert K., Choi D.Y., Liu M., Kim H.C., Cass W.A., Sullivan P.G., Bing G. (2007). Inflammation induces mitochondrial dysfunction and dopaminergic neurodegeneration in the nigrostriatal system. J. Neurochem..

[113] Zhang J., Stanton D.M., Nguyen X.V., Liu M., Zhang Z., Gash D., Bing G. (2005). Intrapallidal lipopolysaccharide injection increases iron and ferritin levels in glia of the rat substantia nigra and induces locomotor deficits. Neuroscience.

[114] Zhang Z., Zhang K., Du X., Li Y. (2012). Neuroprotection of desferrioxamine in lipopolysaccharide-induced nigrostriatal dopamine neuron degeneration. Mol. Med. Rep..

[115] Kim C.Y., Alcalay R.N. (2017). Genetic Forms of Parkinson’s Disease. Semin. Neurol..

[116] Sai Y., Zou Z., Peng K., Dong Z. (2012). The Parkinson’s disease-related genes act in mitochondrial homeostasis. Neurosci. Biobehav. Rev..

[117] Nalls M.A., Plagnol V., Hernandez D.G., Sharma M., Sheerin U.M., Saad M., Simon-Sanchez J., Schulte C., Lesage S., International Parkinson Disease Genomics Consortium (2011). Imputation of sequence variants for identification of genetic risks for Parkinson’s disease: A meta-analysis of genome-wide association studies. Lancet.

[118] Mata I.F., Checkoway H., Hutter C.M., Samii A., Roberts J.W., Kim H.M., Agarwal P., Alvarez V., Ribacoba R., Pastor P. (2012). Common variation in the LRRK2 gene is a risk factor for Parkinson’s disease. Mov. Disord. Off. J. Mov. Disord. Soc..

[119] Moehle M.S., Webber P.J., Tse T., Sukar N., Standaert D.G., DeSilva T.M., Cowell R.M., West A.B. (2012). LRRK2 inhibition attenuates microglial inflammatory responses. J. Neurosci. Off. J. Soc. Neurosci..

[120] Daher J.P., Volpicelli-Daley L.A., Blackburn J.P., Moehle M.S., West A.B. (2014). Abrogation of alpha-synuclein-mediated dopaminergic neurodegeneration in LRRK2-deficient rats. Proc. Natl. Acad. Sci. USA.

[121] Ma B., Xu L., Pan X., Sun L., Ding J., Xie C., Koliatsos V.E., Cai H. (2016). LRRK2 modulates microglial activity through regulation of chemokine (C-X3-C) receptor 1 -mediated signalling pathways. Hum. Mol. Genet..

[122] Van Duijn C.M., Dekker M.C., Bonifati V., Galjaard R.J., Houwing-Duistermaat J.J., Snijders P.J., Testers L., Breedveld G.J., Horstink M., Sandkuijl L.A. (2001). Park7, a novel locus for autosomal recessive early-onset parkinsonism, on chromosome 1p36. Am. J. Hum. Genet..

[123] Bonifati V., Rizzu P., van Baren M.J., Schaap O., Breedveld G.J., Krieger E., Dekker M.C., Squitieri F., Ibanez P., Joosse M. (2003). Mutations in the DJ-1 gene associated with autosomal recessive early-onset parkinsonism. Science.

[124] Chien C.H., Lee M.J., Liou H.C., Liou H.H., Fu W.M. (2016). Microglia-Derived Cytokines/Chemokines Are Involved in the Enhancement of LPS-Induced Loss of Nigrostriatal Dopaminergic Neurons in DJ-1 Knockout Mice. PLoS ONE.

[125] Skelly D.T., Hennessy E., Dansereau M.A., Cunningham C. (2013). A systematic analysis of the peripheral and CNS effects of systemic LPS, IL-1beta, [corrected] TNF-alpha and IL-6 challenges in C57BL/6 mice. PLoS ONE.

[126] Cazareth J., Guyon A., Heurteaux C., Chabry J., Petit-Paitel A. (2014). Molecular and cellular neuroinflammatory status of mouse brain after systemic lipopolysaccharide challenge: Importance of CCR2/CCL2 signaling. J. Neuroinflamm..

[127] Qin L., Wu X., Block M.L., Liu Y., Breese G.R., Hong J.S., Knapp D.J., Crews F.T. (2007). Systemic LPS causes chronic neuroinflammation and progressive neurodegeneration. Glia.

[128] Beier E.E., Neal M., Alam G., Edler M., Wu L.J., Richardson J.R. (2017). Alternative microglial activation is associated with cessation of progressive dopamine neuron loss in mice systemically administered lipopolysaccharide. Neurobiol. Dis..

[129] Liu Y., Qin L., Wilson B., Wu X., Qian L., Granholm A.C., Crews F.T., Hong J.S. (2008). Endotoxin induces a delayed loss of TH-IR neurons in substantia nigra and motor behavioral deficits. Neurotoxicology.

[130] Jangula A., Murphy E.J. (2013). Lipopolysaccharide-induced blood brain barrier permeability is enhanced by alpha-synuclein expression. Neurosci. Lett..

[131] Zheng H.F., Yang Y.P., Hu L.F., Wang M.X., Wang F., Cao L.D., Li D., Mao C.J., Xiong K.P., Wang J.D. (2013). Autophagic impairment contributes to systemic inflammation-induced dopaminergic neuron loss in the midbrain. PLoS ONE.

[132] Czapski G.A., Cakala M., Chalimoniuk M., Gajkowska B., Strosznajder J.B. (2007). Role of nitric oxide in the brain during lipopolysaccharide-evoked systemic inflammation. J. Neurosci. Res..

[133] Qin L., Liu Y., Hong J.S., Crews F.T. (2013). NADPH oxidase and aging drive microglial activation, oxidative stress, and dopaminergic neurodegeneration following systemic LPS administration. Glia.

[134] L’Episcopo F., Tirolo C., Testa N., Caniglia S., Morale M.C., Impagnatiello F., Marchetti B. (2011). Switching the microglial harmful phenotype promotes lifelong restoration of subtantia nigra dopaminergic neurons from inflammatory neurodegeneration in aged mice. Rejuvenation Res..

[135] Zhao Y.F., Qiong Z., Zhang J.F., Lou Z.Y., Zu H.B., Wang Z.G., Zeng W.C., Kai Y., Xiao B.G. (2018). The Synergy of Aging and LPS Exposure in a Mouse Model of Parkinson’s Disease. Aging Dis..

[136] Hudson A.J. (1981). Amyotrophic lateral sclerosis and its association with dementia, parkinsonism and other neurological disorders: A review. Brain J. Neurol..

[137] Charles T., Swash M. (2001). Amyotrophic lateral sclerosis: Current understanding. J. Neurosci. Nurs. J. Am. Assoc. Neurosci. Nurses.

[138] Leigh P.N., Whitwell H., Garofalo O., Buller J., Swash M., Martin J.E., Gallo J.M., Weller R.O., Anderton B.H. (1991). Ubiquitin-immunoreactive intraneuronal inclusions in amyotrophic lateral sclerosis. Morphology, distribution, and specificity. Brain J. Neurol..

[139] Alexianu M.E., Kozovska M., Appel S.H. (2001). Immune reactivity in a mouse model of familial ALS correlates with disease progression. Neurology.

[140] Graves M.C., Fiala M., Dinglasan L.A., Liu N.Q., Sayre J., Chiappelli F., van Kooten C., Vinters H.V. (2004). Inflammation in amyotrophic lateral sclerosis spinal cord and brain is mediated by activated macrophages, mast cells and T cells. Amyotroph. Lateral Scler. Other Mot. Neuron Disord..

[141] Gurney M.E., Pu H., Chiu A.Y., Dal Canto M.C., Polchow C.Y., Alexander D.D., Caliendo J., Hentati A., Kwon Y.W., Deng H.X. (1994). Motor neuron degeneration in mice that express a human Cu,Zn superoxide dismutase mutation. Science.

[142] Valente T., Straccia M., Gresa-Arribas N., Dentesano G., Tusell J.M., Serratosa J., Mancera P., Sola C., Saura J. (2013). CCAAT/enhancer binding protein delta regulates glial proinflammatory gene expression. Neurobiol. Aging.

[143] Ohgomori T., Yamada J., Takeuchi H., Kadomatsu K., Jinno S. (2016). Comparative morphometric analysis of microglia in the spinal cord of SOD1(G93A) transgenic mouse model of amyotrophic lateral sclerosis. Eur. J. Neurosci..

[144] Byrne S., Walsh C., Lynch C., Bede P., Elamin M., Kenna K., McLaughlin R., Hardiman O. (2011). Rate of familial amyotrophic lateral sclerosis: A systematic review and meta-analysis. J. Neurol. Neurosurg. Psychiatry.

[145] Correia A.S., Patel P., Dutta K., Julien J.P. (2015). Inflammation Induces TDP-43 Mislocalization and Aggregation. PLoS ONE.

[146] Lyon M.S., Wosiski-Kuhn M., Gillespie R., Caress J., Milligan C. (2019). Inflammation, Immunity, and amyotrophic lateral sclerosis: I. Etiology and pathology. Muscle Nerve.

[147] Liu J., Wang F. (2017). Role of Neuroinflammation in Amyotrophic Lateral Sclerosis: Cellular Mechanisms and Therapeutic Implications. Front. Immunol..

[148] Huang W.J., Chen W.W., Zhang X. (2016). Huntington’s disease: Molecular basis of pathology and status of current therapeutic approaches. Exp. Ther. Med..

[149] Folstein S.E. (1991). The psychopathology of Huntington’s disease. Res. Publ. Assoc. Res. Nerv. Ment. Dis..

[150] MacDonald M.E., Ambrose C.M., Duyao M.P., Myers R.H., Lin C., Srinidhi L., Barnes G., Taylor S.A., James M., Groot N. (1993). A novel gene containing a trinucleotide repeat that is expanded and unstable on Huntington’s disease chromosomes. Cell.

[151] Labbadia J., Morimoto R.I. (2013). Huntington’s disease: Underlying molecular mechanisms and emerging concepts. Trends Biochem. Sci..

[152] Gunawardena S., Goldstein L.S. (2005). Polyglutamine diseases and transport problems: Deadly traffic jams on neuronal highways. Arch. Neurol..

[153] Li S.H., Li X.J. (2004). Huntingtin-protein interactions and the pathogenesis of Huntington’s disease. Trends Genet. TIG.

[154] Sugars K.L., Rubinsztein D.C. (2003). Transcriptional abnormalities in Huntington disease. Trends Genet. TIG.

[155] Silvestroni A., Faull R.L., Strand A.D., Moller T. (2009). Distinct neuroinflammatory profile in post-mortem human Huntington’s disease. Neuroreport.

[156] Bjorkqvist M., Wild E.J., Thiele J., Silvestroni A., Andre R., Lahiri N., Raibon E., Lee R.V., Benn C.L., Soulet D. (2008). A novel pathogenic pathway of immune activation detectable before clinical onset in Huntington’s disease. J. Exp. Med..

[157] Dalrymple A., Wild E.J., Joubert R., Sathasivam K., Bjorkqvist M., Petersen A., Jackson G.S., Isaacs J.D., Kristiansen M., Bates G.P. (2007). Proteomic profiling of plasma in Huntington’s disease reveals neuroinflammatory activation and biomarker candidates. J. Proteome Res..

[158] Bouchard J., Truong J., Bouchard K., Dunkelberger D., Desrayaud S., Moussaoui S., Tabrizi S.J., Stella N., Muchowski P.J. (2012). Cannabinoid receptor 2 signaling in peripheral immune cells modulates disease onset and severity in mouse models of Huntington’s disease. J. Neurosci. Off. J. Soc. Neurosci..

[159] Franciosi S., Ryu J.K., Shim Y., Hill A., Connolly C., Hayden M.R., McLarnon J.G., Leavitt B.R. (2012). Age-dependent neurovascular abnormalities and altered microglial morphology in the YAC128 mouse model of Huntington disease. Neurobiol. Dis..

[160] Renoir T., Pang T.Y., Shikano Y., Li S., Hannan A.J. (2015). Loss of the Sexually Dimorphic Neuro-Inflammatory Response in a Transgenic Mouse Model of Huntington’s Disease. J. Huntington’s Dis..

[161] Kempuraj D., Thangavel R., Natteru P.A., Selvakumar G.P., Saeed D., Zahoor H., Zaheer S., Iyer S.S., Zaheer A. (2016). Neuroinflammation Induces Neurodegeneration. J. Neurol. Neurosurg. Spine.

[162] Glass C.K., Saijo K., Winner B., Marchetto M.C., Gage F.H. (2010). Mechanisms underlying inflammation in neurodegeneration. Cell.

[163] Liu M., Bing G. (2011). Lipopolysaccharide animal models for Parkinson’s disease. Parkinson’s Dis..

[164] Okun E., Griffioen K.J., Lathia J.D., Tang S.C., Mattson M.P., Arumugam T.V. (2009). Toll-like receptors in neurodegeneration. Brain Res. Rev..

[165] Schmalz G., Krifka S., Schweikl H. (2011). Toll-like receptors, LPS, and dental monomers. Adv. Dent. Res..

[166] Madera-Salcedo I.K., Cruz S.L., Gonzalez-Espinosa C. (2013). Morphine prevents lipopolysaccharide-induced TNF secretion in mast cells blocking IkappaB kinase activation and SNAP-23 phosphorylation: Correlation with the formation of a beta-arrestin/TRAF6 complex. J. Immunol..

[167] Lee S.J., Lee S. (2002). Toll-like receptors and inflammation in the CNS. Curr. Drug Targets Inflamm. Allergy.

[168] Tak P.P., Firestein G.S. (2001). NF-kappaB: A key role in inflammatory diseases. J. Clin. Investig..

[169] Bachstetter A.D., Xing B., de Almeida L., Dimayuga E.R., Watterson D.M., Van Eldik L.J. (2011). Microglial p38alpha MAPK is a key regulator of proinflammatory cytokine up-regulation induced by toll-like receptor (TLR) ligands or beta-amyloid (Abeta). J. Neuroinflamm..

[170] Francois A., Terro F., Janet T., Rioux Bilan A., Paccalin M., Page G. (2013). Involvement of interleukin-1beta in the autophagic process of microglia: Relevance to Alzheimer’s disease. J. Neuroinflamm..

[171] Wu D., Zhang X., Zhao M., Zhou A.L. (2015). The role of the TLR4/NF-kappaB signaling pathway in Abeta accumulation in primary hippocampal neurons. Sheng Li Xue Bao Acta Physiol. Sin..

[172] McMillian M., Kong L.Y., Sawin S.M., Wilson B., Das K., Hudson P., Hong J.S., Bing G. (1995). Selective killing of cholinergic neurons by microglial activation in basal forebrain mixed neuronal/glial cultures. Biochem. Biophys. Res. Commun..

[173] Yang X., Zhang J.D., Duan L., Xiong H.G., Jiang Y.P., Liang H.C. (2018). Microglia activation mediated by toll-like receptor-4 impairs brain white matter tracts in rats. J. Biomed. Res..

[174] Hoozemans J.J., Veerhuis R., Janssen I., van Elk E.J., Rozemuller A.J., Eikelenboom P. (2002). The role of cyclo-oxygenase 1 and 2 activity in prostaglandin E(2) secretion by cultured human adult microglia: Implications for Alzheimer’s disease. Brain Res..

[175] Shi J., Johansson J., Woodling N.S., Wang Q., Montine T.J., Andreasson K. (2010). The prostaglandin E2 E-prostanoid 4 receptor exerts anti-inflammatory effects in brain innate immunity. J. Immunol..

[176] Huang Y.Y., Zhang Q., Zhang J.N., Zhang Y.N., Gu L., Yang H.M., Xia N., Wang X.M., Zhang H. (2018). Triptolide up-regulates metabotropic glutamate receptor 5 to inhibit microglia activation in the lipopolysaccharide-induced model of Parkinson’s disease. Brain Behav. Immun..

[177] Barger S.W., Chavis J.A., Drew P.D. (2000). Dehydroepiandrosterone inhibits microglial nitric oxide production in a stimulus-specific manner. J. Neurosci. Res..

[178] Dewil M., dela Cruz V.F., Van Den Bosch L., Robberecht W. (2007). Inhibition of p38 mitogen activated protein kinase activation and mutant SOD1(G93A)-induced motor neuron death. Neurobiol. Dis..

[179] Jeohn G.H., Cooper C.L., Wilson B., Chang R.C., Jang K.J., Kim H.C., Liu B., Hong J.S. (2002). p38 MAP kinase is involved in lipopolysaccharide-induced dopaminergic neuronal cell death in rat mesencephalic neuron-glia cultures. Ann. N. Y. Acad. Sci..

[180] Fiebich B.L., Butcher R.D., Gebicke-Haerter P.J. (1998). Protein kinase C-mediated regulation of inducible nitric oxide synthase expression in cultured microglial cells. J. Neuroimmunol..

[181] Akundi R.S., Candelario-Jalil E., Hess S., Hull M., Lieb K., Gebicke-Haerter P.J., Fiebich B.L. (2005). Signal transduction pathways regulating cyclooxygenase-2 in lipopolysaccharide-activated primary rat microglia. Glia.

[182] Bauer M.K., Lieb K., Schulze-Osthoff K., Berger M., Gebicke-Haerter P.J., Bauer J., Fiebich B.L. (1997). Expression and regulation of cyclooxygenase-2 in rat microglia. Eur. J. Biochem..

[183] Yousif N.M., de Oliveira A.C.P., Brioschi S., Huell M., Biber K., Fiebich B.L. (2018). Activation of EP2 receptor suppresses poly(I: C) and LPS-mediated inflammation in primary microglia and organotypic hippocampal slice cultures: Contributing role for MAPKs. Glia.

[184] Bronstein D.M., Perez-Otano I., Sun V., Mullis Sawin S.B., Chan J., Wu G.C., Hudson P.M., Kong L.Y., Hong J.S., McMillian M.K. (1995). Glia-dependent neurotoxicity and neuroprotection in mesencephalic cultures. Brain Res..

[185] Gayle D.A., Ling Z., Tong C., Landers T., Lipton J.W., Carvey P.M. (2002). Lipopolysaccharide (LPS)-induced dopamine cell loss in culture: Roles of tumor necrosis factor-alpha, interleukin-1beta, and nitric oxide. Brain Res. Dev. Brain Res..

[186] Zhu Y., Chen X., Liu Z., Peng Y.P., Qiu Y.H. (2016). Interleukin-10 Protection against Lipopolysaccharide-Induced Neuro-Inflammation and Neurotoxicity in Ventral Mesencephalic Cultures. Int. J. Mol. Sci..

[187] Zhang F., Qian L., Flood P.M., Shi J.S., Hong J.S., Gao H.M. (2010). Inhibition of IkappaB kinase-beta protects dopamine neurons against lipopolysaccharide-induced neurotoxicity. J. Pharmacol. Exp. Ther..

[188] Bhat N.R., Zhang P., Lee J.C., Hogan E.L. (1998). Extracellular signal-regulated kinase and p38 subgroups of mitogen-activated protein kinases regulate inducible nitric oxide synthase and tumor necrosis factor-alpha gene expression in endotoxin-stimulated primary glial cultures. J. Neurosci. Off. J. Soc. Neurosci..

[189] Cuenda A., Rousseau S. (2007). p38 MAP-kinases pathway regulation, function and role in human diseases. Biochim. Biophys. Acta.

[190] Gordon R., Singh N., Lawana V., Ghosh A., Harischandra D.S., Jin H., Hogan C., Sarkar S., Rokad D., Panicker N. (2016). Protein kinase Cdelta upregulation in microglia drives neuroinflammatory responses and dopaminergic neurodegeneration in experimental models of Parkinson’s disease. Neurobiol. Dis..

[191] Bhatia H.S., Roelofs N., Munoz E., Fiebich B.L. (2017). Alleviation of Microglial Activation Induced by p38 MAPK/MK2/PGE2 Axis by Capsaicin: Potential Involvement of other than TRPV1 Mechanism/s. Sci. Rep..

[192] Fiebich B.L., Lieb K., Engels S., Heinrich M. (2002). Inhibition of LPS-induced p42/44 MAP kinase activation and iNOS/NO synthesis by parthenolide in rat primary microglial cells. J. Neuroimmunol..

[193] Fiebich B.L., Schleicher S., Butcher R.D., Craig A., Lieb K. (2000). The neuropeptide substance P activates p38 mitogen-activated protein kinase resulting in IL-6 expression independently from NF-kappa B. J. Immunol..

[194] Anantharam V., Kitazawa M., Wagner J., Kaul S., Kanthasamy A.G. (2002). Caspase-3-dependent proteolytic cleavage of protein kinase Cdelta is essential for oxidative stress-mediated dopaminergic cell death after exposure to methylcyclopentadienyl manganese tricarbonyl. J. Neurosci. Off. J. Soc. Neurosci..

[195] Kaul S., Kanthasamy A., Kitazawa M., Anantharam V., Kanthasamy A.G. (2003). Caspase-3 dependent proteolytic activation of protein kinase C delta mediates and regulates 1-methyl-4-phenylpyridinium (MPP+)-induced apoptotic cell death in dopaminergic cells: Relevance to oxidative stress in dopaminergic degeneration. Eur. J. Neurosci..

[196] Zhang D., Anantharam V., Kanthasamy A., Kanthasamy A.G. (2007). Neuroprotective effect of protein kinase C delta inhibitor rottlerin in cell culture and animal models of Parkinson’s disease. J. Pharmacol. Exp. Ther..

[197] Zhang W., Gao J.H., Yan Z.F., Huang X.Y., Guo P., Sun L., Liu Z., Hu Y., Zuo L.J., Yu S.Y. (2018). Minimally Toxic Dose of Lipopolysaccharide and alpha-Synuclein Oligomer Elicit Synergistic Dopaminergic Neurodegeneration: Role and Mechanism of Microglial NOX2 Activation. Mol. Neurobiol..

[198] Kim C., Lv G., Lee J.S., Jung B.C., Masuda-Suzukake M., Hong C.S., Valera E., Lee H.J., Paik S.R., Hasegawa M. (2016). Exposure to bacterial endotoxin generates a distinct strain of alpha-synuclein fibril. Sci. Rep..

[199] Arai H., Furuya T., Yasuda T., Miura M., Mizuno Y., Mochizuki H. (2004). Neurotoxic effects of lipopolysaccharide on nigral dopaminergic neurons are mediated by microglial activation, interleukin-1beta, and expression of caspase-11 in mice. J. Biol. Chem..

